# Statistical Inference for Heterogeneous Competing Risks Model Under Improved Adaptive Type-II Progressive Censoring

**DOI:** 10.3390/e28060609

**Published:** 2026-05-28

**Authors:** Junrui Wang

**Affiliations:** School of Finance, Lanzhou University of Finance and Economics, Lanzhou 730020, China; wangjunrui@lzufe.edu.cn

**Keywords:** heterogeneous competing risk, improve adaptive Type II progressive censored sample, bootstrap, Markov Chain Monte Carlo

## Abstract

This study investigates statistical inference for a heterogeneous competing risks model under an improved adaptive Type-II progressive censoring scheme, which effectively controls testing time while ensuring sufficient failure data. Assuming the latent lifetimes of distinct failure causes are independent and follow Chen and Weibull distributions, we develop both frequentist and Bayesian approaches to derive point and interval estimates for the unknown parameters. Interval estimators include approximate confidence intervals, bootstrap confidence intervals, and highest posterior density credible intervals. Under the Bayesian framework, Markov Chain Monte Carlo techniques are utilized to obtain numerical solutions under the squared error loss function, assuming independent gamma priors. Extensive Monte Carlo simulations and a real-world data application are presented to demonstrate the efficacy and practical utility of the proposed methodologies.

## 1. Introduction

The statistical modeling and inference of system reliability based on product failure data constitutes a fundamental research area within reliability theory and reliability engineering. As product performance continues to improve, obtaining complete failure data from life-testing experiments has become increasingly difficult; consequently, to reduce experimental duration and associated costs, censored data are typically collected instead of complete observations. Among the censoring schemes (CS) most commonly employed in reliability studies are the Type-I CS (T-I CS), Type-II CS (T-II CS), and Type-I/Type-II hybrid CS (T-I(II) HCS); see, e.g., [1,2,3,4,5,6,7,8,9]. These schemes, however, share a notable limitation: they do not permit the withdrawal of test units prior to the termination of the experiment, which is at odds with many practical scenarios. In medical follow-up studies, for instance, participants may drop out partway through the trial owing to unavoidable circumstances. To overcome this drawback, progressive censoring schemes (PCSs)—including the progressive Type-I CS (T-I PCS) and progressive Type-II CS (T-II PCS)—have been introduced, under which test units may be removed at predetermined stages in accordance with a pre-specified removal scheme. Such schemes not only curtail experimental costs but also shorten the overall testing duration; see, e.g., [10,11,12,13].

Recent advances in science and technology have given rise to a growing number of highly reliable, long-lifetime products, presenting both novel challenges and opportunities for statistical inference in system reliability. In this context, existing PCSs often prove impractical owing to the potentially prolonged duration of the associated life tests. By integrating the T-II PCS with the HCS, Kundu and Joarder [14] proposed Type-II progressive hybrid censoring scheme (T-II PHCS) for given test time threshold *T*, which effectively solves the problem of longer test time, but cannot avoid the problem of smaller sample size of obtained test data. To defeat this limitation, Ng et al. [15] introduced an adaptive Type-II progressive censored scheme (AT-II PCS) to ensure that there were enough test data samples when the trial time slightly exceeded *T* by adjusting the preset removal scheme without changing the total number of removed individuals. As demonstrated by Ng et al. [15], the AT-II PCS method proves to be efficient in parameter estimation when the total test duration is not of significant concern. Nevertheless, in cases where the test units are highly reliable products, the experiment duration can become quite lengthy, and the AT-II PCS may not guarantee a satisfactory total test duration. Recently, Yan et al. [16] addressed this challenge by giving two time thresholds $T_{1},T_{2}\left(T_{1}<T_{2}\right),$ and introducing a new CS called the improved adaptive progressive Type-II censoring scheme (IAT-II PCS). This scheme has two advantages: First of all, under the premise of obtaining enough test data, the experiment can be effectively ensured to end within a predetermined time. Secondly, it includes several special deletion schemes, such as AT-II PCS, T-II PHCS, and T-II CS. Therefore, the IAT-II PCS method is recommended when the reliability engineer wants to end the test within the specified time.

In practice, a system in operation is often subject to multiple potential failure factors that act competitively—a phenomenon known as competing risks—whereby the occurrence of one failure factor precludes the manifestation of the others. Consequently, studies of competing failures typically focus on the first failure event. In recent years, within the fields of reliability theory and reliability engineering, competing risks models have been extensively investigated under a variety of CSs and lifetime distribution assumptions. For example, Hudgens et al. [17] derived maximum likelihood estimate (MLE) for cumulative incidence function with interval-censored competing risk data. Wu and Shi [18] tackled Bayesian estimation using T-I PHCS and binomial removals from a two-parameter Gompertz distribution. Jin and Lai [19] innovated regression analysis for censored competing risks data, directly modeling cumulative incidence function and using iterative method for parameter estimation. Other studies encompass AT-II PCS for independent exponential risk factors [20], Weibull competing risk models based on HCS [21], Chen competing risk models with T-II PCS [13], step-stress accelerated dependent competing risk models [22], T-I PCS for generalized inverted exponential distributions [23], Weibull competing risks based on generalized T-II PHCS [24], and generalized linear exponential competing risks models with T-II PCS [25]. By contrast, comparatively few studies have addressed competing risks data under the IAT-II PCS framework. Dutta and Kayal [26] derived the MLE of parameters of exponential distribution for independent competing risks model under IAT-II PCS. Elshahhat and Nassar [27] studies the inference of the competing risks model from Weibull distribution under IAT-II PCS. Dutta and Kayal [28] derived the MLE of parameters of exponential distribution for independent competing risks model under IAT-II PCS. Elshahhat and Nassar [27] studied the inference of the competing risks model from Weibull distribution under IAT-II PCS. Alotaibi et al. [29] investigated the estimation of inverted Weibull competing risks model using improved adaptive progressive Type-II censoring plan. Dey and Kayal [30] examined the statistical inference of Chen lifetime competing risks model based on improved adaptive Type-II progressive censored data. The bulk of the existing literature on competing risks, however, rests on the homogeneity assumption—namely, that all competing failure factors follow distributions from the same parametric family. In many practical settings, the underlying failure mechanisms of different competing factors differ substantially, and consequently their lifetime distributions need not belong to a common family. For example, Ranjan et al. [31] and Ranjan and Upadhyay [32] introduced Bayesian analysis and MLE for Gamma-Exponential competing risk models. Recent work by Tarvirdizade and Ahmadpour [33] introduced Chen–Weibull (C–W) distribution, with minimum Chen and Weibull distributions, which is very flexible to model the bathtub-shaped hazard rate data and its hazard rate function is simple. Abba et al. [34] present MLE and Bayesian analysis of flexible additive Chen-Gompertz distribution. Such distributions are well suited to the modeling of heterogeneous competing risks.

To the best of our knowledge, no existing study has investigated parameter estimation for heterogeneous competing risks data under the IAT-II PCS. To fill this gap, the present work makes the following contributions. First, an IAT-II PCS heterogeneous competing risks model is formulated on the basis of the C–W distribution under the assumption of independent competing risk factors. Second, both maximum likelihood estimation (MLE) and Bayesian approaches are employed to derive point and interval estimates of the model parameters; specifically, three types of interval estimates are considered—approximate confidence intervals (ACIs), Bootstrap confidence intervals (Bootstrap-CIs), and highest posterior density (HPD) credible intervals. For the Bayesian procedure in particular, following Yousaf et al. [35] and Ren and Gui [36], the Gamma distribution is adopted as the prior, and numerical solutions for the parameter estimates are obtained via the Markov Chain Monte Carlo (MCMC) algorithm under the squared error loss (SEL) function. Finally, the proposed methods are assessed through Monte Carlo simulations and further illustrated with a real-data application.

The remainder of this paper is organized as follows. Section 2 introduces the preliminary concepts and the underlying model assumptions. Section 3 presents the MLE based on the Newton–Raphson (NR) method, together with the ACIs and Bootstrap-CIs for the model parameters. Section 4 develops the Bayesian estimation and HPD CIs for the unknown parameters using the MCMC method with Gibbs sampling. Section 5 reports a Monte Carlo simulation study, with the relevant tables provided in the Appendix B. A real-data analysis is presented in Section 6. Finally, Section 7 concludes the paper with a summary of the main findings.

## 2. Data and Model Assumption

Consider a lifetime experiment with *n* identical units, with lifetimes defined by independent and identically distributed (i.i.d.) random variables $X_{1},X_{2},\ldots ,X_{n}.$ Without loss of generality, assume that each unit is exposed to only two competing risk factors, that is, $X_{i}=\min\left\{X_{1i},X_{2i}\right\}$ for i=1,2,…,n, where $X_{ki},k=1,2,$ represents the latent failure time of the *i*-th unit under the *k*-th cause of failure. Furthermore, we assume that the latent failure times $X_{1i}$ and $X_{2i}$ are heterogeneous and independent following the Chen and Weibull distributions, respectively. Then, for all $x>0,\alpha_{1}>0,\beta_{1}>0,\alpha_{2}>0,\beta_{2}>0,$ the cumulative distribution function (CDF) and the probability density function (PDF) of $X_{1i}$ can be expressed as

(1) $$\begin{array}{cc} F_{1}\left(x;\alpha_{1},\beta_{1}\right) & =1-e^{\alpha_{1}\left(1-e^{x^{\beta_{1}}}\right)},\\ f_{1}\left(x;\alpha_{1},\beta_{1}\right) & =\alpha_{1}\beta_{1}x^{\beta_{1}-1}e^{x^{\beta_{1}}}e^{\alpha_{1}\left(1-e^{x^{\beta_{1}}}\right)}, \end{array}$$

respectively, and the CDF and PDF of $X_{2i}$ can be expressed as

(2) $$\begin{array}{cc} F_{2}\left(x;\alpha_{2},\beta_{2}\right) & =1-e^{-\alpha_{2}x^{\beta_{2}}},\\ f_{2}\left(x;\alpha_{2},\beta_{2}\right) & =\alpha_{2}\beta_{2}x^{\beta_{2}-1}e^{-\alpha_{2}x^{\beta_{2}}}, \end{array}$$

respectively, then, the CDF and PDF of $X_{i}$ can be expressed as

(3) $$\begin{array}{cc} F(x,\alpha_{1},\beta_{1},\alpha_{2},\beta_{2}) & =1-e^{\alpha_{1}\left(1-e^{x^{\beta_{1}}}\right)-\alpha_{2}x^{\beta_{2}}},\\ f(x,\alpha_{1},\beta_{1},\alpha_{2},\beta_{2}) & =\left(\alpha_{1}\beta_{1}x^{\beta_{1}-1}e^{x^{\beta_{1}}}+\alpha_{2}\beta_{2}x^{\beta_{2}-1}\right)e^{\alpha_{1}\left(1-e^{x^{\beta_{1}}}\right)-\alpha_{2}x^{\beta_{2}}}, \end{array}$$

respectively.

**Figure 1:**
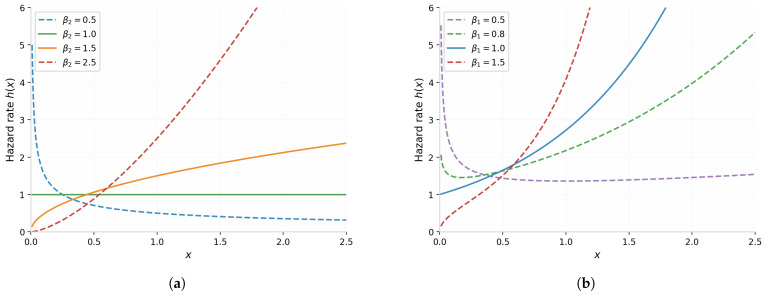
Hazard rate functions of the Weibull distribution (**a**) and Chen distribution (**b**) for different shape parameters and $\alpha_{1}=\alpha_{2}=1$.

The choice of this heterogeneous pairing is motivated by three considerations. First, the two families offer complementary hazard-shape flexibility, the Weibull hazard is monotone—increasing for $\beta_{2}>1$, decreasing for $\beta_{2}<1$, and constant for $\beta_{2}=1$—thereby capturing the standard wear-out, infant-mortality, and memoryless patterns commonly seen in reliability data, whereas the Chen distribution additionally accommodates bathtub-shaped hazards (when $\beta_{1}<1$) that arise in systems subject to high initial failure intensity, a quiescent intermediate phase, and increasing late-life failures. These complementary shapes are illustrated in Figure 1: the Figure 1a displays the strict monotonicity of the Weibull hazard, while the Figure 1b reveals the bathtub minima of the Chen hazard at $\beta_{1}=0.5$ and $\beta_{1}=0.8$ (marked with symbols), so that together the two families span the principal hazard patterns cataloged in lifetime data analysis. Second, both distributions possess closed-form CDFs, which allows the joint sub-density of competing-risks observations under the IAT-II PCS scheme to be expressed in closed form as well—an advantage that the lognormal and inverse Gaussian, whose CDFs involve special functions, do not share under heavy censoring. Third, in the actual data analysis, we found that using the Chen distribution and the Weibull distribution to fit the failure data caused by failure cause 1 and failure cause 2, respectively, and validating through the Kolmogorov–Smirnov (KS) test, both distributions showed high fitting accuracy, which further confirms the reasonableness of the model specification.

Suppose an experiment involving a set of *n* units, having independent lifetime $X_{1},X_{2},\ldots ,X_{n}$, it is assumption that for *i*-th unit (i=1,2,…,n), its lifetime is represented as $X_{i}=\min\left(X_{1i},X_{2i}\right)$, where, $X_{1i}∼Chen\left(\alpha_{1},\beta_{1}\right)$ and $X_{2i}∼Weibull\left(\alpha_{2},\beta_{2}\right)$. Before commencing the experiment, two critical pieces of information are given: the number of failures to be observed, denoted as *m*, and a predefined PCS $R=(R_{1},R_{2},\ldots ,R_{m-1}$,$R_{m}=n-m-\sum_{i=1}^{m-1}R_{i})$, with $R_{i}\geq 0.$ During the experiment, when the *i*-th unit experiences a failure, and its lifetime is denoted as $X_{i:m:n}$, we remove a specified number of units, which is determined by $R_{i}$, from the remaining units in the experiment. It is worth noting that the value of $R_{i}$ may be adjusted as the experiment progresses. Additionally, two predetermined threshold values, $T_{1}$ and $T_{2}$ with $0<T_{1}<T_{2}<\infty$, are set in advance. $T_{1}$ serves as the first threshold, acting as a warning regarding the testing duration. When the experiment reaches $T_{1}$, it indicates the need to accelerate the testing process. The experiment can continue beyond this point. $T_{2}$ functions as the second threshold, signifying the maximum allowable duration for the experiment. Regardless of whether the desired number of failures (*m*) has been reached or not, the experiment must be terminated once it reaches $T_{2}$. This experiment is called IAT-II PCS, a detailed explanation is given the following:

- If $X_{m:m:n}<T_{1}$, the experiment ends before time $T_{1}$, resembling the Type-II PCS with the censoring plan $R=(R_{1},R_{2},\ldots ,R_{m})$.
- If $T_{1}<X_{m:m:n}<T_{2}$, where $k_{1}$ is such that $X_{k_{1}:m:n}<T_{1}<X_{(k_{1}+1):m:n}$, the experiment concludes at $X_{m:m:n}$, akin to the AT-II PCS with the censoring plan $R=(R_{1},R_{2},\ldots ,R_{k_{1}},0,0,\ldots ,0,n-m-\sum_{i=1}^{k_{1}}R_{i})$.
- If $X_{m:m:n}$ exceeds the experiment time allowed by $T_{2}$, where $k_{2}\;\left(k_{1}\leq k_{2}<m\right)$ is such that $X_{k_{2}:m:n}<T_{2}<X_{(k_{2}+1):m:n}$, the experiment ends at $T_{2}$, resembling the IAT-II PCS with the censoring plan $R=(R_{1},R_{2},\ldots ,R_{k_{1}},0,0,\ldots ,0,n-k_{2}-\sum_{i=1}^{k_{1}}R_{i})$.

**Figure 2:**
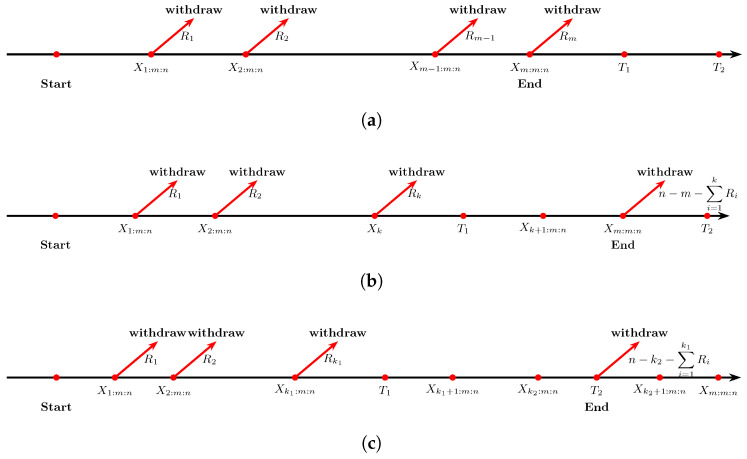
(**a**) Experiment terminates before time $T_{1}$ (i.e., $X_{m:m:n}<T_{1}$). (**b**) Experiment terminates between times $T_{1}$ and $T_{2}$ (i.e., $T_{1}\leq X_{m:m:n}\leq T_{2}$). (**c**) Experiment terminates after time $T_{2}$ (i.e., $X_{m:m:n}\geq T_{2}>T_{1}$). Improve adaptive Type II progressive censoring scheme.

A schematic illustration of the IAT-II PCS procedure is provided in Figure 2; further details can be found in Yan et al. [16].

Based on the IAT-II PCS competing risk data, as illustrated in Figure 2, we observe as follows:

(4) $$\begin{array}{c} \{\left(X_{i:m:n},R_{i},\delta_{i}\right){|}_{i=1,2\ldots ,n_{2}},\left(X_{j:m:n},0,\delta_{j}\right){|}_{j=n_{2}+1,n_{2}+2\ldots ,n_{1}-1},\left(X_{n_{1}:m:n},R_{n_{1}},\delta_{n_{1}}\right),\left(t^{*},R^{*},\delta^{*}\right)\}, \end{array}$$

where $\delta_{l}=1,2$ denotes that unit *l* failed at time $X_{l:m:n}\;\left(l=1,2,\ldots ,n_{1}\right)$ because of the first and second causes of failures, respectively. Let $t^{*}=\min\left\{X_{m:m:n},T_{2}\right\}$ denotes the time of termination of the test. In addition, $R_{n_{1}}=R^{*}\cdot I\left(t^{*}=X_{m:m:n}\right)$, $\delta^{*}=\delta_{n_{1}}\cdot I\left(t^{*}=X_{m:m:n}\right)$, $k_{1}<k_{2}<m$, $T_{1}<T_{2}$, and

(n1,n2,R*)={(5a)(m,m,n−m−∑i=1m−1Ri),Xm:m:n<T1,(5b)(m,k1,n−m−∑i=1k1Ri),Xk1:m:n<T1<Xk1+1:m:n,Xm:m:n<T2,(5c)(k2,k1,n−k2−∑i=1k1Ri),Xk1:m:n<T1<Xk1+1:m:n,Xk2:m:n<T2<Xk2+1:m:n,

where

- Equation (5a) denotes the T-II PCS;
- Equation (5b) denotes the AT-II PCS;
- Equation (5c) denotes the IAT-II PCS.

Let

(6) $$I(\delta_{l}=k)=\{\begin{array}{c} 1,\;\delta_{l}=k,\\ 0,\;\delta_{l}\neq k, \end{array}$$

where l=1,2,⋯,m, then, the random variables $m_{1}=\sum_{i=1}^{m}I\left(\delta_{i}=1\right)$ and $m_{2}=\sum_{i=1}^{m}I$$\left(\delta_{i}=2\right)$ describe the number of failures due to the first and second cause of failures, respectively. Therefore, $m_{1}+m_{2}=m$, in which *m* is considered positive and fixed.

Using the independence of the latent failure times $X_{1i}$ and $X_{2i},i=1,\cdots ,n$, we obtain the relative risk rate of failure cause 1 can be obtained by calculating

(7) $$\begin{array}{c} q=P\left(X_{1i}\leq X_{2i}\right)=\alpha_{2}\beta_{2}\int_{0}^{\infty }x^{\beta_{2}-1}e^{-\alpha_{2}x^{\beta_{2}}}\left(1-e^{\alpha_{1}\left(1-e^{x^{\beta_{1}}}\right)}\right)dx, \end{array}$$

and we have $m_{1}∼\mathrm{Binomial}\left(m,q\right)$ and $m_{2}∼\mathrm{Binomial}\left(m,1-q\right)$.

For convenience, sample $\left(X_{1:m:n},X_{2:m:n},\ldots ,X_{n_{1}:m:n},t^{*}\right)$ is abbreviated as $\left(x_{1},x_{2},\ldots ,x_{n_{1}},t^{*}\right)$. From data (4), we can write the likelihood function in the presence of the IAT-II PCS based on competing risks as follows:

(8) $$\begin{array}{ccc} L(x) & = & C\prod_{i=1}^{n_{1}}{\left[f_{1}\left(x_{i}\right)S_{2}\left(x_{i}\right)\right]}^{I(\delta_{i}=1)}{\left[f_{2}\left(x_{i}\right)S_{1}\left(x_{i}\right)\right]}^{I(\delta_{i}=2)}\prod_{i=1}^{n_{2}}{\left[S_{1}\left(x_{i}\right)S_{2}\left(x_{i}\right)\right]}^{R_{i}}{\left[S_{1}\left(t^{*}\right)S_{2}\left(t^{*}\right)\right]}^{R^{*}}, \end{array}$$

where $C=\prod_{i=1}^{n_{1}}\left(n-i+1-\sum_{j=1}^{\min(j-1,n_{2})}\right)$ is a constant that does not depend on the parameters $S_{k}\left(x\right)=1-F_{k}\left(x\right),k=1,2.$

## 3. Frequentist Inference

In this section, we investigate the MLE, together with the ACIs and Bootstrap-CIs, for the IAT-II PCS in the presence of heterogeneous competing risks data.

### 3.1. Maximum Likelihood Estimation

To derive the MLEs of the unknown parameters, substituting (1) and (2) into (8) yields the likelihood function of the observed data as follows:

(9) $$\begin{array}{ccc} L(\alpha_{1},\alpha_{2},\beta_{1},\beta_{2}|x) & \propto & {\alpha_{1}}^{m_{1}}{\beta_{1}}^{m_{1}}{\alpha_{2}}^{m_{2}}{\beta_{2}}^{m_{2}}\prod_{i=1}^{n_{1}}{x_{i}}^{\sum_{k=1}^{2}\left(\beta_{k}-1\right)I\left(\delta_{i}=k\right)}\\ & & \times \exp\{\sum_{i=1}^{n_{1}}{x_{i}}^{\beta_{1}}I\left(\delta_{i}=1\right)-\sum_{k=1}^{2}\alpha_{k}\eta_{k}\left(\beta_{k}\right)\}, \end{array}$$

where $\eta_{1}\left(\beta_{1}\right)=\sum_{i=1}^{n_{1}}\left(e^{{x_{i}}^{\beta_{1}}}-1\right)+\sum_{i=1}^{n_{2}}R_{i}\left(e^{{x_{i}}^{\beta_{1}}}-1\right)+R^{*}\left(e^{{t^{*}}^{\beta_{1}}}-1\right)$ and $\eta_{2}\left(\beta_{2}\right)=\sum_{i=1}^{n_{1}}{x_{i}}^{\beta_{2}}+\sum_{i=1}^{n_{2}}R_{i}{x_{i}}^{\beta_{2}}+R^{*}{t^{*}}^{\beta_{2}}$.

Furthermore, the associated log-likelihood function is given by

(10) $$\begin{array}{ccc} l(\alpha_{1},\alpha_{2},\beta_{1},\beta_{2}|x) & \propto & \sum_{k=1}^{2}m_{k}\log\alpha_{k}+\sum_{k=1}^{2}m_{k}\log\beta_{k}+\sum_{i=1}^{n_{1}}\sum_{k=1}^{2}\left(\beta_{k}-1\right)I\left(\delta_{i}=k\right)\log x_{i}\\ & & +\sum_{i=1}^{n_{1}}{x_{i}}^{\beta_{1}}I\left(\delta_{i}=1\right)-\sum_{k=1}^{2}\alpha_{k}\eta_{k}\left(\beta_{k}\right). \end{array}$$

Notably, for k=1,2,

$$\begin{array}{ccc} \frac{\partial l(\alpha_{1},\alpha_{2},\beta_{1},\beta_{2}|x)}{\partial \alpha_{k}} & \propto & \frac{m_{k}}{\alpha_{k}}-\eta_{k}\left(\beta_{k}\right). \end{array}$$

Thus, we can obtain the following estimator of $\alpha_{k}:$

$$\begin{array}{ccc} {\hat{\alpha }}_{k} & = & \frac{m_{k}}{\eta_{k}\left(\beta_{k}\right)}. \end{array}$$

By substituting ${\hat{\alpha }}_{k}$ into (10), the profile likelihood functions of $\beta_{1}$ and $\beta_{2}$ can be expressed as follows:

(11) $$\begin{array}{ccc} l_{PL}\left(\beta_{1},\beta_{2}|x\right) & = & \underset{\alpha_{1},\alpha_{2}\in \Theta }{\sup}l\left(\alpha_{1},\alpha_{2},\beta_{1},\beta_{2}|x\right)\\ & = & l({\hat{\alpha }}_{1},{\hat{\alpha }}_{2},\beta_{1},\beta_{2}|x)\\ & \propto & -\sum_{k=1}^{2}m_{k}\log\eta \left(\beta_{k}\right)+\sum_{k=1}^{2}m_{k}\log\beta_{k}+\sum_{i=1}^{n_{1}}\sum_{k=1}^{2}{x_{i}}^{\beta_{k}}I\left(\delta_{i}=k\right)\\ & & +\sum_{i=1}^{n_{1}}\left(\beta_{1}-1\right)I\left(\delta_{i}=1\right)\log x_{i}\\ & = & l_{1}\left(\beta_{1}\right)+l_{2}\left(\beta_{2}\right), \end{array}$$

where

(12) $$\begin{array}{ccc} l_{1}\left(\beta_{1}\right) & = & -m_{1}\log\eta_{1}\left(\beta_{1}\right)+m_{1}\log\beta_{1}+\sum_{i=1}^{n_{1}}{x_{i}}^{\beta_{1}}I\left(\delta_{i}=1\right)\\ & & +\sum_{i=1}^{n_{1}}\left(\beta_{1}-1\right)I\left(\delta_{i}=1\right)\log x_{i} \end{array}$$

and

(13) $$\begin{array}{ccc} l_{2}\left(\beta_{2}\right) & = & -m_{2}\log\eta_{2}\left(\beta_{2}\right)+m_{2}\log\beta_{2}+\sum_{i=1}^{n_{1}}\left(\beta_{2}-1\right)I\left(\delta_{i}=2\right)\log x_{i}. \end{array}$$

By taking derivative in (12) and (13) with respect to $\beta_{1}$ and $\beta_{2},$ respectively, and making them equal to zero, we obtain

(14) $$\begin{array}{ccc} \frac{\partial l_{1}\left(\beta_{1}\right)}{\partial \beta_{1}} & = & -m_{1}\frac{\eta_{1}^{\prime }\left(\beta_{1}\right)}{\eta_{1}\left(\beta_{1}\right)}+\frac{m_{1}}{\beta_{1}}+\sum_{i=1}^{n_{1}}\left(x_{i}^{\beta_{1}}+1\right)I\left(\delta_{i}=1\right)\log x_{i}=0 \end{array}$$

and

(15) $$\begin{array}{ccc} \frac{\partial l_{2}\left(\beta_{2}\right)}{\partial \beta_{2}} & = & -m_{2}\frac{\eta_{2}^{\prime }\left(\beta_{2}\right)}{\eta_{2}\left(\beta_{2}\right)}+\frac{m_{2}}{\beta_{2}}+\sum_{i=1}^{n_{1}}I\left(\delta_{i}=2\right)\log x_{i}=0, \end{array}$$

where

(16) $$\begin{array}{ccc} \eta_{1}^{\prime }\left(\beta_{1}\right) & = & \sum_{i=1}^{n_{1}}x_{i}^{\beta_{1}}e^{x_{i}^{\beta_{1}}}\log x_{i}+\sum_{i=1}^{n_{2}}R_{i}x_{i}^{\beta_{1}}e^{x_{i}^{\beta_{1}}}\log x_{i}+R^{*}t^{*\beta_{1}}e^{t^{*\beta_{1}}}\log t^{*} \end{array}$$

and

(17) $$\begin{array}{ccc} \eta_{2}^{\prime }\left(\beta_{2}\right) & = & \sum_{i=1}^{n_{2}}x_{i}^{\beta_{2}}\log x_{i}+\sum_{i=1}^{n_{2}}R_{i}x_{i}^{\beta_{2}}\log x_{i}+R^{*}t^{*\beta_{1}}\log t^{*}. \end{array}$$

From (14), the MLE of $\beta_{1}$ can be obtained by solving the following nonlinear equation:

$$\begin{array}{ccc} H_{1}\left(\beta_{1},x\right) & = & \beta_{1}, \end{array}$$

where

(18) $$\begin{array}{ccc} H_{1}\left(\beta_{1},x\right) & = & {\left[\frac{\eta_{1}^{\prime }\left(\beta_{1}\right)}{\eta_{1}\left(\beta_{1}\right)}-\frac{1}{m_{1}}\sum_{i=1}^{n_{1}}\left(x_{i}^{\beta_{1}}+1\right)I\left(\delta_{i}=1\right)\log x_{i}\right]}^{-1}. \end{array}$$

Similarly, from (15), the MLE of $\beta_{2}$ can be obtained by solving the following nonlinear equation:

$$\begin{array}{ccc} H_{2}\left(\beta_{2},x\right) & = & \beta_{2}, \end{array}$$

where

(19) $$\begin{array}{ccc} H_{2}\left(\beta_{2},x\right) & = & {\left[\frac{\eta_{2}^{\prime }\left(\beta_{2}\right)}{\eta_{2}\left(\beta_{2}\right)}-\frac{1}{m_{2}}\sum_{i=1}^{n_{1}}I\left(\delta_{i}=1\right)\log x_{i}\right]}^{-1}. \end{array}$$

Clearly, the nonlinear Equations (18) and (19) do not admit closed-form solutions for $\beta_{1}$ and $\beta_{2}$, respectively; consequently, numerical iterative procedures such as the NR method must be employed to obtain the MLEs of $\beta_{1}$ and $\beta_{2}$. The following theorem establishes that the likelihood Equation (14) admits a unique solution. The verification of uniqueness for the likelihood Equation (15) proceeds analogously to that of Lemma 3 in [36] and is therefore omitted. The result below formally states the existence and uniqueness of the MLE for the parameter $\beta_{1}$.

**Theorem** **1.** *The MLE of $\beta_{1}$ derived from Equation (16) not only exists but also remains unique for $0<x_{i}\leq 1$*.

**Proof.**  See Appendix A.    □

### 3.2. Asymptotic Confidence Intervals

In this subsection, CIs for $\alpha_{1},\alpha_{2},\beta_{1},$ and $\beta_{2}$ are constructed using the asymptotic normality property of MLE. Let $\theta =(\alpha_{1},\alpha_{2},\beta_{1},\beta_{2}).$ Using (10), (16) and (17), the Fisher information matrix of θ can be expressed as follows:

(20) $$I\left(\theta \right)=\left(\begin{array}{cccc} I_{11} & I_{12} & I_{13} & I_{14}\\ I_{21} & I_{22} & I_{23} & I_{24}\\ I_{31} & I_{32} & I_{33} & I_{34}\\ I_{41} & I_{42} & I_{43} & I_{44} \end{array}\right),$$

where the associated elements are

$$\begin{array}{ccc} I_{11} & = & -\frac{\partial^{2}l}{\partial \alpha_{1}^{2}}=\frac{m_{1}}{\alpha_{1}^{2}},I_{12}=-\frac{\partial^{2}l}{\partial \alpha_{1}\partial \alpha_{2}}=0,I_{13}=-\frac{\partial^{2}l}{\partial \alpha_{1}\partial \beta_{1}}=\eta_{1}^{\prime }\left(\beta_{1}\right),I_{14}=-\frac{\partial^{2}l}{\partial \alpha_{1}\partial \beta_{2}}=0,\\ I_{22} & = & -\frac{\partial^{2}l}{\partial \alpha_{2}^{2}}=\frac{m_{2}}{\alpha_{2}^{2}},I_{23}=-\frac{\partial^{2}l}{\partial \alpha_{2}\partial \beta_{1}}=0,I_{24}=-\frac{\partial^{2}l}{\partial \alpha_{2}\partial \beta_{2}}=\eta_{2}^{\prime }\left(\beta_{2}\right),I_{34}=-\frac{\partial^{2}l}{\partial \beta_{1}\partial \beta_{2}}=0,\\ I_{33} & = & -\frac{\partial^{2}l}{\partial \beta_{1}^{2}}=\frac{m_{1}}{\beta_{1}^{2}}-\sum_{i=1}^{n_{1}}x_{i}^{\beta_{1}}{\left(\log x_{i}\right)}^{2}+\alpha_{1}\eta_{1}^{\prime \prime }\left(\beta_{1}\right),I_{44}=-\frac{\partial^{2}l}{\partial \beta_{2}^{2}}=\frac{m_{2}}{\beta_{2}^{2}}+\alpha_{2}\eta_{2}^{\prime \prime }\left(\beta_{2}\right), \end{array}$$

with

(21) $$\begin{array}{ccc} \eta_{1}^{\prime \prime }\left(\beta_{1}\right) & = & \sum_{i=1}^{n_{1}}x_{i}^{\beta_{1}}\left(x_{i}^{\beta_{1}}+1\right)e^{x_{i}^{\beta_{1}}}{\left(\log x_{i}\right)}^{2}+\sum_{i=1}^{n_{2}}R_{i}x_{i}^{\beta_{1}}\left(x_{i}^{\beta_{1}}+1\right)e^{x_{i}^{\beta_{1}}}{\left(\log x_{i}\right)}^{2}\\ & & +R^{*}t^{*\beta_{1}}\left(t^{*\beta_{1}}+1\right)e^{t^{*\beta_{1}}}{\left(\log t^{*}\right)}^{2} \end{array}$$

and

$$\begin{array}{ccc} \eta_{2}^{\prime \prime }\left(\beta_{2}\right) & = & \sum_{i=1}^{n_{2}}x_{i}^{\beta_{2}}{\left(\log x_{i}\right)}^{2}+\sum_{i=1}^{n_{2}}R_{i}x_{i}^{\beta_{2}}{\left(\log x_{i}\right)}^{2}+R^{*}t^{*\beta_{1}}{\left(\log t^{*}\right)}^{2}. \end{array}$$

Using the asymptotic distribution of MLE, we have that $\hat{\theta }\overset{d}{⟶}N\left(\theta ,I^{-1}\left(\theta \right)\right),$ where $I^{-1}\left(\theta \right)$ is the inverse of matrix I(θ) and

$$I^{-1}\left(\hat{\theta }\right)=I^{-1}\left(\theta \right){|}_{\theta =\hat{\theta }}=\left(\begin{array}{cccc} var(\hat{\alpha_{1}}) & Cov(\hat{\alpha_{1}},\hat{\alpha_{2}}) & Cov(\hat{\alpha_{1}},\hat{\beta_{1}}) & Cov(\hat{\alpha_{1}},\hat{\beta_{2}})\\ Cov(\hat{\alpha_{2}},\hat{\alpha_{2}}) & var(\hat{\alpha_{2}}) & Cov(\hat{\alpha_{2}},\hat{\beta_{1}}) & Cov(\hat{\alpha_{2}},\hat{\beta_{2}})\\ Cov(\hat{\beta_{1}},\hat{\alpha_{1}}) & Cov(\hat{\beta_{1}},\hat{\alpha_{2}}) & var(\hat{\beta_{1}}) & Cov(\hat{\beta_{1}},\hat{\beta_{2}})\\ Cov(\hat{\beta_{2}},\hat{\alpha_{1}}) & Cov(\hat{\beta_{2}},\hat{\alpha_{2}}) & Cov(\hat{\beta_{2}},\hat{\beta_{1}}) & var(\hat{\beta_{2}}) \end{array}\right).$$

For 0≤γ≤1, the 100(1−γ)% ACIs of $\theta_{1}=\alpha_{1},\theta_{2}=\alpha_{2},\theta_{3}=\beta_{1},$ and $\theta_{4}=\beta_{2}$ are given by

$$\left(\hat{\theta_{i}}-z_{\gamma /2}var\left(\hat{\theta_{i}}\right),\hat{\theta_{i}}+z_{\gamma /2}var\left(\hat{\theta_{i}}\right)\right),\;i=1,2,3,4.$$

It is noteworthy that for i=1,2,3,4,$\hat{\theta_{i}}-z_{\gamma /2}var\left(\hat{\theta_{i}}\right)\leq 0$ may be established. This contradicts the fact that $\theta_{i}>0.$ Thus, the log transformation of ${\hat{\theta }}_{i}$ can overcome this drawback. Using log-transformation and delta method, we have

$$\log{\hat{\theta }}_{i}\overset{d}{⟶}N\left(\log\theta_{i},var({\hat{\theta }}_{i})/{\hat{\theta }}_{i}^{2}\right),\;i=1,2,3,4.$$

Then, the modify 100(1−γ)% ACIs are

$$\left(\frac{{\hat{\theta }}_{i}}{\exp\left(z_{\gamma /2}\sqrt{var\left({\hat{\theta }}_{i}\right)/{\hat{\theta }}_{i}^{2}}\right)},{\hat{\theta }}_{i}\exp\left(z_{\gamma /2}\sqrt{var\left({\hat{\theta }}_{i}\right)/{\hat{\theta }}_{i}^{2}}\right)\right),i=1,2,3,4.$$

### 3.3. Bootstrap Confidence Intervals

It is well known that the construction of ACIs for unknown parameters via the asymptotic distribution of the MLE relies on large-sample properties, and is therefore no longer applicable when the sample size is relatively small. To address this issue, in this subsection we propose to construct parametric Bootstrap-CIs via the bootstrap method. Specifically, we consider two bootstrap procedures—the boot-p and boot-t methods—introduced by [37,38], respectively.

Using these procedures, we construct CIs based on the 100(γ/2)-th and 100(1−γ/2)-th quantiles of the empirical bootstrap distribution. The detailed implementations are given as follows: Algorithm 1 describes the Bootstrap-p procedure, and Algorithm 2 describes the Bootstrap-t procedure.
**Algorithm 1** Bootstrap-p Algorithm**Require:** 
$n,m,R,T_{1},T_{2},\gamma$, and initial values $\alpha_{1}^{0},\beta_{1}^{0},\alpha_{2}^{0},\beta_{2}^{0},\theta^{0}$.**Ensure:** 
Bootstrap-p confidence interval.  1:Generate sample data D=(x,δ), and compute the maximum likelihood estimates (MLEs) of model parameters $\hat{\theta }=\left({\hat{\alpha }}_{1},{\hat{\alpha }}_{2},{\hat{\beta }}_{1},{\hat{\beta }}_{2}\right)$;  2:Based on the MLEs $\hat{\theta }$ of model parameters, combined with $n,m,R,T_{1},T_{2}$, generate a set of Bootstrap samples $\left(x_{11}^{*},x_{12}^{*},\ldots ,x_{1m}^{*}\right)$;  3:Based on the generated Bootstrap samples, compute the MLEs of model parameters:$${\hat{\theta }}^{*(1)}=\left({\hat{\alpha }}_{1}^{*(1)},{\hat{\alpha }}_{2}^{*(1)},{\hat{\beta }}_{1}^{*(1)},{\hat{\beta }}_{2}^{*(1)}\right);$$  4:Repeat Step 3 for *B* times, and obtain *B* sets of MLEs of model parameters:$${\hat{\theta }}^{*(b)}=\left({\hat{\alpha }}_{1}^{*(b)},{\hat{\alpha }}_{2}^{*(b)},{\hat{\beta }}_{1}^{*(b)},{\hat{\beta }}_{2}^{*(b)}\right),\;b=1,2,\ldots ,B;$$  5:Sort the *B* sets of MLEs in ascending order to obtain $\left({\hat{\theta }}^{*[1]},{\hat{\theta }}^{*[2]},\ldots ,{\hat{\theta }}^{*[B]}\right)$, where:$${\hat{\theta }}^{*[b]}=\left({\hat{\alpha }}_{1}^{*[b]},{\hat{\alpha }}_{2}^{*[b]},{\hat{\beta }}_{1}^{*[b]},{\hat{\beta }}_{2}^{*[b]}\right),\;b=1,2,\ldots ,B;$$  6:Given the confidence level 100(1−γ)%, obtain the two-sided Bootstrap-p confidence intervals for model parameters:$$\left({\hat{\alpha }}_{1}^{*[\frac{B\gamma }{2}]},{\hat{\alpha }}_{1}^{*[B\left(1-\frac{\gamma }{2}\right)]}\right),\left({\hat{\alpha }}_{2}^{*[\frac{B\gamma }{2}]},{\hat{\alpha }}_{2}^{*[B\left(1-\frac{\gamma }{2}\right)]}\right),\left({\hat{\beta }}_{1}^{*[\frac{B\gamma }{2}]},{\hat{\beta }}_{1}^{*[B\left(1-\frac{\gamma }{2}\right)]}\right),\left({\hat{\beta }}_{2}^{*[\frac{B\gamma }{2}]},{\hat{\beta }}_{2}^{*[B\left(1-\frac{\gamma }{2}\right)]}\right).$$

**Algorithm 2** Bootstrap-t Algorithm
**Require:** 
$n,m,R,T_{1},T_{2},k,\gamma$, and initial values $\alpha_{1}^{0},\beta_{1}^{0},\alpha_{2}^{0},\beta_{2}^{0},\theta^{0}$.**Ensure:** 
Bootstrap-t confidence interval.  1:The first 3 steps are the same as those in Algorithm 1, and are omitted here;  2:Construct the T-statistics related to model parameters:$$T_{k}^{*(1)}=\frac{{\hat{\alpha }}_{k}^{*(1)}-{\hat{\alpha }}_{k}}{\sqrt{\mathrm{var}({\hat{\alpha }}_{k}^{*(1)})}},\;T_{k+2}^{*(1)}=\frac{{\hat{\beta }}_{k}^{*(1)}-{\hat{\beta }}_{k}}{\sqrt{\mathrm{var}({\hat{\beta }}_{k}^{*(1)})}},\;k=1,2;$$  3:Repeat the above steps *B* times, and obtain $T^{*(b)}=\left(T_{1}^{*(b)},T_{2}^{*(b)},T_{3}^{*(b)},T_{4}^{*(b)}\right)$, b=1,2,…,B;  4:Sort $T^{*(1)},T^{*(2)},\ldots ,T^{*(B)}$ in ascending order to obtain $T^{*[1]},T^{*[2]},\ldots ,T^{*[B]}$;  5:Given the confidence level 100(1−γ)%, obtain the two-sided Bootstrap-t confidence intervals for model parameters:$$\left({\hat{\alpha }}_{k}+T_{k}^{*[\frac{B\gamma }{2}]}\sqrt{\mathrm{var}({\hat{\alpha }}_{k})},\;{\hat{\alpha }}_{k}+T_{k}^{*[B\left(1-\frac{\gamma }{2}\right)]}\sqrt{\mathrm{var}({\hat{\alpha }}_{k})}\right),\;k=1,2,$$$$\left({\hat{\beta }}_{k}+T_{k+2}^{*[\frac{B\gamma }{2}]}\sqrt{\mathrm{var}({\hat{\beta }}_{k})},\;{\hat{\beta }}_{k}+T_{k+2}^{*[B\left(1-\frac{\gamma }{2}\right)]}\sqrt{\mathrm{var}({\hat{\beta }}_{k})}\right),\;k=1,2.$$


## 4. Bayesian Inferences

The Bayesian approach offers several advantages over the MLE approach for statistical inference. In particular, it allows prior information about the parameters to be incorporated into subsequent analysis: when such prior information is available, the Bayesian framework yields a posterior distribution that combines the prior with the observed data, on the basis of which reliable inferences can be drawn even from small datasets. In this section, we develop Bayesian estimation of the parameter vector $\left(\alpha_{1},\alpha_{2},\beta_{1},\beta_{2}\right)$ for the heterogeneous competing risks model under the IAT-II PCS lifetime test, with the Bayes estimates obtained under the SEL function via the Gibbs sampling algorithm.

Following [35,36,39], the parameter pairs $\left(\alpha_{1},\beta_{1}\right)$ and $\left(\alpha_{2},\beta_{2}\right)$ are assumed to follow independent gamma distributions. It is readily seen that the prior distribution

$$\pi \left(\alpha_{1}\right)=G\left(a_{1},b_{1}\right)\propto \left\{\begin{array}{cc} \alpha_{1}^{a_{1}-1}e^{-b_{1}\alpha_{1}}, & \mathrm{if}\;\alpha_{1}>0,\\ 0, & \mathrm{if}\;\alpha_{1}\leq 0. \end{array}\right.,$$

$$\pi \left(\beta_{1}\right)=G\left(a_{3},b_{3}\right)\propto \left\{\begin{array}{cc} \beta_{1}^{a_{3}-1}e^{-b_{3}\beta_{1}}, & \mathrm{if}\;\beta_{1}>0,\\ 0, & \mathrm{if}\;\beta_{1}\leq 0. \end{array}\right.,$$

$$\pi \left(\alpha_{2}\right)=G\left(a_{2},b_{2}\right)\propto \left\{\begin{array}{cc} \alpha_{2}^{a_{2}-1}e^{-b_{2}\alpha_{2}}, & \mathrm{if}\;\alpha_{2}>0,\\ 0, & \mathrm{if}\;\alpha_{2}\leq 0. \end{array}\right.,$$

and

$$\pi \left(\beta_{2}\right)=G\left(a_{4},b_{4}\right)\propto \left\{\begin{array}{cc} \beta_{2}^{a_{4}-1}e^{-b_{4}\beta_{2}}, & \mathrm{if}\;\beta_{2}>0,\\ 0, & \mathrm{if}\;\beta_{2}\leq 0. \end{array}\right.$$

where all hyperparameters $a_{i},b_{i},\;i=1,2,3,4$ are specifically known and positive.

Therefore, for $\alpha_{1},\alpha_{2},\beta_{1},\beta_{2},$ the joint prior distribution is given by

$$\begin{array}{ccc} \pi (\alpha_{1},\alpha_{2},\beta_{1},\beta_{2}) & = & \pi \left(\alpha_{1}\right)\pi \left(\alpha_{2}\right)\pi \left(\beta_{1}\right)\pi \left(\beta_{2}\right)\\ & = & \prod_{i=1}^{2}\alpha_{i}^{a_{i}-1}e^{-\sum_{i=1}^{2}b_{i}\alpha_{i}}\prod_{k=3}^{4}\beta_{k-2}^{a_{k}-1}e^{-\sum_{i=1}^{2}b_{k}\beta_{k-2}}. \end{array}$$

Subsequently, the joint posterior distribution can be written as follows:

(22) $$\begin{array}{cc} \pi (\alpha_{1},\alpha_{2},\beta_{1},\beta_{2}|x)\; & =\frac{L\left(\alpha_{1},\alpha_{2},\beta_{1},\beta_{2},x\right)\pi \left(\alpha_{1},\alpha_{2},\beta_{1},\beta_{2}\right)}{\int_{0}^{\infty }\;\;\;\int_{0}^{\infty }\;\;\;\int_{0}^{\infty }\;\;\;\int_{0}^{\infty }L\left(\alpha_{1},\alpha_{2},\beta_{1},\beta_{2},x\right)\pi \left(\alpha_{1},\alpha_{2},\beta_{1},\beta_{2}\right)\;d\alpha_{1}d\alpha_{2}d\beta_{1}d\beta_{2}}\\ \; & \propto \alpha_{1}^{m_{1}+a_{1}-1}\alpha_{2}^{m_{2}+a_{2}-1}\beta_{1}^{m_{1}+a_{3}-1}\beta_{2}^{m_{2}+a_{4}-1}\prod_{i=1}^{n_{1}}x_{i}^{\sum_{k=1}^{2}\left(\beta_{k}-1\right)I\left(\delta_{i}=k\right)}\\ \; & \;\times \exp\left\{-\sum_{i=1}^{n_{1}}x_{i}^{\beta_{1}}I\left(\delta_{i}=1\right)-\sum_{k=1}^{2}\alpha_{k}\left(b_{k}+\eta_{k}\left(\beta_{k}\right)\right)-b_{3}\beta_{1}-b_{4}\beta_{2}\right\}. \end{array}$$

Assume that ν is a function of $\alpha_{1},\alpha_{2},\beta_{1},$ and $\beta_{2},$ using the SEL function, the Bayesian estimator of $\nu (\alpha_{1},\alpha_{2},\beta_{1},\beta_{2})$ can be expressed as the posterior mean:

(23) $${\hat{\nu }}_{B}=\frac{\int_{0}^{\infty }\;\;\;\int_{0}^{\infty }\;\;\;\int_{0}^{\infty }\;\;\;\int_{0}^{\infty }\nu L\left(\alpha_{1},\alpha_{2},\beta_{1},\beta_{2},x\right)\pi \left(\alpha_{1},\alpha_{2},\beta_{1},\beta_{2}\right)\;d\alpha_{1}d\alpha_{2}d\beta_{1}d\beta_{2}}{\int_{0}^{\infty }\;\;\;\int_{0}^{\infty }\;\;\;\int_{0}^{\infty }\;\;\;\int_{0}^{\infty }L\left(\alpha_{1},\alpha_{2},\beta_{1},\beta_{2},x\right)\pi \left(\alpha_{1},\alpha_{2},\beta_{1},\beta_{2}\right)\;d\alpha_{1}d\alpha_{2}d\beta_{1}d\beta_{2}}.$$

Note that the ratio of integrals (23) cannot be expressed in closed form. Thus, we adopt the MCMC method to compute the Bayesian estimator.

Gibbs sampling is an extensively applied technique for generating samples from the full conditional probability distribution to compute Bayesian estimates and construct the HPD CIs. The Gibbs sampling algorithm is a well-known method for constructing Markov chains, it calculates the probability of the next sample as a conditional probability given the prior sample. For this purpose, (22) can be written as

$$\begin{array}{ccc} \pi_{k}\left(\alpha_{k}|\beta_{k}\right) & = & \alpha_{k}^{m_{k}+a_{k}-1}e^{-\alpha_{k}\left(b_{k}+\eta_{k}\left(\beta_{k}\right)\right)},\;k=1,2,\\ \pi_{3}\left(\beta_{1}|\alpha_{1}\right) & = & \beta_{1}^{m_{1}+a_{3}-1}\prod_{i=1}^{n_{1}}x_{i}^{\left(\beta_{1}-1\right)I\left(\delta_{i}=1\right)}\exp\left(-\sum_{i=1}^{n_{1}}x_{i}^{\beta_{1}}I\left(\delta_{i}=1\right)-\alpha_{1}\eta_{1}\left(\beta_{1}\right)-b_{3}\beta_{1}\right),\\ \pi_{4}\left(\beta_{2}|\alpha_{2}\right) & = & \beta_{2}^{m_{2}+a_{4}-1}\prod_{i=1}^{n_{1}}x_{i}^{\left(\beta_{2}-1\right)I\left(\delta_{i}=2\right)}\exp\left(-\alpha_{2}\eta_{2}\left(\beta_{2}\right)-b_{4}\beta_{2}\right). \end{array}$$

The Metropolis–Hastings (MH) algorithm is often used with the Gibbs sampling procedure to obtain Bayesian estimation and HPD CIs, which is a viable method for constructing an MCMC chain. The Bayesian simulation process is conducted based on Algorithm 3. To assess MCMC sampler convergence, we run 3 parallel chains. Then, the posterior means are obtained as follows:

$${\hat{\nu }}_{B}=\frac{\sum_{j=1}^{3}\sum_{i=A+1}^{M}\nu_{j}^{\left(i\right)}}{3(M-A)},$$

where *M* denotes the number of numerical simulations and *A* denotes the number of burn-in periods.
**Algorithm 3** M–H within Gibbs sampler for Bayesian estimation and HPD credible interval construction under the IAT-II PCS competing-risks model.  1:Given the IAT-II PCS sample $(x_{1},x_{2},\ldots ,x_{m}),$ the prior hyper-parameters ${\left\{\left(a_{k},b_{k}\right)\right\}}_{k=1}^{4},$ the initial values $(\alpha_{1}^{\left(0\right)},\beta_{1}^{\left(0\right)},\alpha_{2}^{\left(0\right)},\beta_{2}^{\left(0\right)}),$ the proposal standard deviation σ>0, the total number of iterations M, the burn-in length A, and acceptance counters $k_{3}=k_{4}=0;$  2:For t=1,2,…,M, repeat Steps 3–7;  3:(Gibbs step for $\alpha_{1}$.) Recognizing $\pi_{1}\left(\alpha_{1}|\beta_{1}^{\left(t-1\right)}\right)$ as a Gamma density, draw $\alpha_{1}^{\left(t\right)}$ directly from$$\alpha_{1}^{\left(t\right)}∼\mathrm{Gamma}\;\left(m_{1}+a_{1},\;b_{1}+\eta_{1}\left(\beta_{1}^{\left(t-1\right)}\right)\right);$$  4:(Gibbs step for $\alpha_{2}$.) Similarly, draw$$\alpha_{2}^{\left(t\right)}∼\mathrm{Gamma}\;\left(m_{2}+a_{2},\;b_{2}+\eta_{2}\left(\beta_{2}^{\left(t-1\right)}\right)\right);$$  5:(M–H step for $\beta_{1}$.) Propose $\beta_{1}^{*}∼N\left(\beta_{1}^{\left(t-1\right)},\sigma^{2}\right).$ If $\beta_{1}^{*}\leq 0,$ automatically reject by setting $\beta_{1}^{\left(t\right)}=\beta_{1}^{\left(t-1\right)};$ otherwise, compute the acceptance probability$$\rho_{1}=\min\left\{1,\;\frac{\pi_{3}\left(\beta_{1}^{*}|\alpha_{1}^{\left(t\right)}\right)}{\pi_{3}\left(\beta_{1}^{\left(t-1\right)}|\alpha_{1}^{\left(t\right)}\right)}\right\},$$
draw U∼U(0,1). If $U\leq \rho_{1},$ accept by setting $\beta_{1}^{\left(t\right)}=\beta_{1}^{*}$ and increment $k_{3}\leftarrow k_{3}+1;$ otherwise set $\beta_{1}^{\left(t\right)}=\beta_{1}^{\left(t-1\right)};$  6:(M–H step for $\beta_{2}$.) Analogous to Step 5 with the target density $\pi_{4}\left(\beta_{2}|\alpha_{2}^{\left(t\right)}\right),$ counter $k_{4},$ and proposal standard deviation σ (possibly tuned separately);  7:Compute $\nu^{\left(t\right)}$ based on $(\alpha_{1}^{\left(t\right)},\alpha_{2}^{\left(t\right)},\beta_{1}^{\left(t\right)},\beta_{2}^{\left(t\right)});$  8:Discard the first *A* iterations as burn-in and retain the post-burn-in sample $\{\nu^{\left(A+1\right)},\nu^{\left(A+2\right)},\ldots ,\nu^{\left(M\right)}\};$  9:Repeat Steps 1–8 three times with dispersed starting values to obtain three independent chains, then compute the Bayes estimate$${\hat{\nu }}_{B}=\frac{\sum_{j=1}^{3}\sum_{i=A+1}^{M}\nu_{j}^{\left(i\right)}}{3(M-A)};$$10:For a fixed j=1,2,3, sort $\left(\nu_{j}^{\left(A+1\right)},\nu_{j}^{\left(A+2\right)},\ldots ,\nu_{j}^{\left(M\right)}\right)$ in ascending order to obtain the order statistics $(\nu_{j}^{\left[A+1\right]},\nu_{j}^{\left[A+2\right]},\ldots ,\nu_{j}^{\left[M\right]}).$ Then, the 100(1−γ)% HPD credible interval is given by$$\min_{l(I_{s})}\left\{I_{s}\right\},$$
where $I_{s}=\left(\nu_{j}^{\left[s\right]},\nu_{j}^{\left[s+(1-\gamma )M\right]}\right),$ A+1≤s≤γM, and $l(I_{s})$ is the length of $I_{s};$11:Output the empirical acceptance rates $k_{3}/M$ and $k_{4}/M$ for the two M–H updates.

## 5. Simulation Experiments

In this section, we conduct a Monte Carlo simulation to investigate the performance of the MLE and Bayesian estimate of IAT-II PCS heterogeneous competing risk data. We consider two criteria for evaluating the estimator’s behavior: the average bias (AB), which is given by ${\mathrm{Bias}}_{{\hat{\theta }}_{i}}=\sum_{j=1}^{N}\left({\hat{\theta }}_{i,j}-\theta_{i}\right)/N,$ and the mean squared error (MSE), which is given by ${\mathrm{MSE}}_{{\hat{\theta }}_{i}}=\sum_{j=1}^{N}{\left({\hat{\theta }}_{i,j}-\theta_{i}\right)}^{2}/N,$ where *N* denotes the number of estimates (i.e., the maximum number of iterations). Additionally, we compute the interval length (IL) and coverage probability (CP). Good estimators should have a bias, approximately zero MSE, short IL, and approximately 0.95 CP. The proposed simulation design agrees with the following setup: the real values of the parameters are chosen as $(\alpha_{1}=0.2,\alpha_{2}=0.4,\beta_{1}=0.6,\beta_{2}=0.8).$ We take $(T_{1}=0.8,T_{2}=1.2),$ $(T_{1}=0.8,T_{2}=1.5),$ $(T_{1}=1,T_{2}=1.5),$ $(T_{1}=0.8,T_{2}=100),$ and $(T_{1}=100,T_{2}=101).$ Using $n,m,T_{1},$ and $T_{2},$ the various CSs are considered, include left censoring (L), right censoring (R), uniform censoring (U), and middle censoring (M), as shown in Table 1.

Next, we present the algorithm (Algorithm 4) to obtain IAT-II PCS heterogeneous competing risk data.
**Algorithm 4** IAT-II PCS heterogeneous competing risk data.1:Given n,m,R and $\left(\alpha_{1},\alpha_{2},\beta_{1},\beta_{2}\right)$, based on R function: **rType2()**, we generate progressive Type II censored data of heterogeneous competing risk denoted by $(X_{1:m:n},X_{2:m:n},\ldots ,X_{m:m:n});$2:By leveraging the relative failure risk rate and the observation that $m_{1}$ follows a Binomial(m,q) distribution, we can derive a set of simulated failure causes denoted as $\left(\delta_{1},\delta_{2},\cdots ,\delta_{m}\right)$;3:Given the time point $T_{1}$ and $T_{2}$, where $T_{1}<T_{2},$ use Step 1 to determine the value of $k_{1},k_{2},$ IAT-II PCS sample can be obtained in one of the following steps;4:if $T_{1}>X_{m:m:n},$ then IAT-II PCS degrades into a Type II progressive censored scheme. Thus, the sample is $(X_{1:m:n},X_{2:m:n},\ldots ,X_{m:m:n}),$ and failure causes is also $\left(\delta_{1},\delta_{2},\cdots ,\delta_{m}\right);$5:if $X_{k_{1}:m:n}<T_{1}<X_{k_{1}+1:m:n}$ and $T_{2}>X_{m:m:n},$ we record $k_{1}.$ Then, IAT-II PCS degrades into an adaptive Type II progressive censored scheme, and we replace the sample $\left(X_{k_{1}+2:m:n},X_{k_{1}+3:m:n},\ldots ,X_{m:m:n}\right)$ with the first $m-k_{1}-1$ order statistics from a truncated PDF $\frac{f(x)}{1-F(X_{k_{1}+1:m:n})}$ with a sample size $(n-k_{1}-1-\sum_{i=1}^{k_{1}}R_{i}),$ where f(x) and F(x) are given in (3). Thus, the sample is$$(X_{1:m:n},X_{2:m:n},\ldots ,X_{k_{1}:m:n},X_{k_{1}+1:m:n},X_{k_{1}+2:m:n}^{*}\ldots ,X_{m:m:n}^{*}),$$
and failure causes is also $\left(\delta_{1},\delta_{2},\cdots ,\delta_{m}\right)$6:if $X_{k_{1}:m:n}<T_{1}<X_{k_{1}+1:m:n}$ and $X_{k_{2}:m:n}<T_{2}<X_{k_{2}+1:m:n},$ we record $k_{1},k_{2}$ and discard sample $(X_{k_{2}+1:m:n}^{*}\ldots ,X_{m:m:n}^{*}).$ Thus, the sample is$$(X_{1:m:n},X_{2:m:n},\ldots ,X_{k_{1}:m:n},X_{k_{1}+1:m:n},X_{k_{1}+2:m:n}^{*}\ldots ,X_{k_{2}:m:n}^{*}),$$
and failure causes is $\left(\delta_{1},\delta_{2},\cdots ,\delta_{k_{2}}\right);$7:if $X_{k_{1}:m:n}<T_{1}<T_{2}<X_{k_{1}+1:m:n},$ that is, $k_{1}=k_{2},$ we record $k_{1}$ and discard the sample $\left(X_{k_{1}+1:m:n}^{*}\ldots ,X_{m:m:n}^{*}\right)$. Thus, the sample is $(X_{1:m:n},X_{2:m:n},\ldots ,X_{k_{1}:m:n}),$ and failure causes is $\left(\delta_{1},\delta_{2},\cdots ,\delta_{k_{1}}\right).$

In our simulation study, the MLEs of the model parameters are computed via the Newton–Raphson (NR) algorithm, with the maximum number of iterations set to M= 10,000 and the convergence tolerance fixed at 0.05. The Bayesian estimators are obtained using MCMC methods. The hyperparameters of the Gamma priors are specified as follows: under the informative scenario, $\left(a_{1},a_{2},a_{3},a_{4}\right)=\left(0.2,0.4,0.6,0.8\right)$ and $\left(b_{1},b_{2},b_{3},b_{4}\right)=\left(1,1,1,1\right)$; under the non-informative scenario, $a_{i}=b_{i}=0.001$ for i=1,2,3,4. Setting $\left(a_{i},b_{i}\right)$ to a small common value such as (0.001,0.001) yields a nearly flat density whose limiting form as $a_{i},b_{i}\to 0$ coincides with the Jeffreys-type non-informative prior π(θ)∝1/θ for a scale parameter (Berger and Bernardo [40]), thereby encoding minimal prior information while ensuring posterior propriety. Moreover, the Gamma prior has been widely adopted in recent Bayesian analyses of survival and competing-risks data under progressive censoring (Yousaf et al. [35], Ren and Gui [36] and Kundu and Gupta [39]), which facilitates direct comparison with existing studies.

In particular, for the MH algorithm, we set the total number of iterations and the burn-in length to M= 10,000 and A= 1000, respectively. The Gaussian random-walk proposal scale is fixed at σ=0.5 throughout the simulations. Across all censoring schemes, the empirical acceptance (Acc) rates fell within the [0.23,0.44] window recommended for one-dimensional random-walk MH updates (W.R. [41]) in 78.3% of the $\beta_{1}$ updates and 92.5% of the $\beta_{2}$ updates. The effective sample size (ESS) of each posterior chain is evaluated by means of Geyer’s initial monotone sequence estimator (Geyer [42]), as implemented in the R (Version 4.5.3) package coda, yielding average ESS values of 1583 for $\beta_{1}$ (minimum ESS =664) and 1842 for $\beta_{2}$ (minimum ESS =1346), both comfortably exceeding the threshold of 400 commonly regarded as sufficient for reliable posterior inference (VATS et al. [43]). Detailed values of the acceptance rates and ESS for each censoring scheme are reported in Table A11, Table A12 and Table A13.

**Figure 3:**
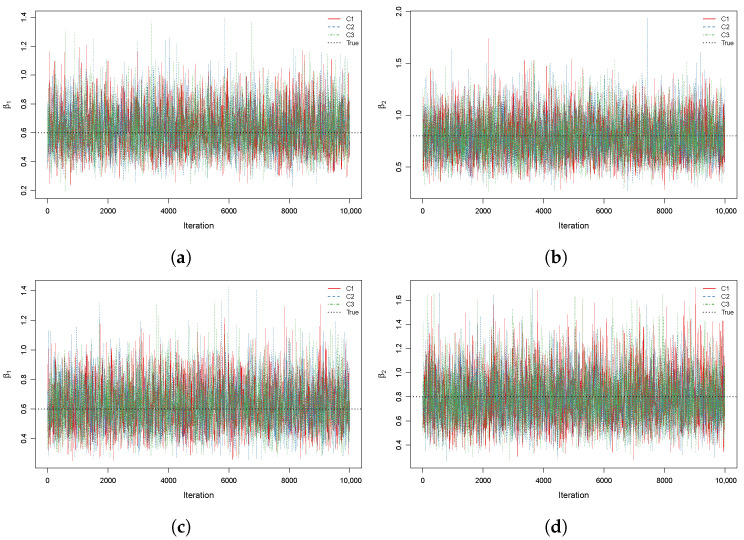
MCMC diagnostics with n=80, m=30, T=(1,1.5). (**a**) Trace plot of $\beta_{1}$ under informative priors. (**b**) Trace plot of $\beta_{2}$ under informative priors. (**c**) Trace plot of $\beta_{1}$ under non-informative priors. (**d**) Trace plot of $\beta_{2}$ under non-informative priors.

**Figure 4:**
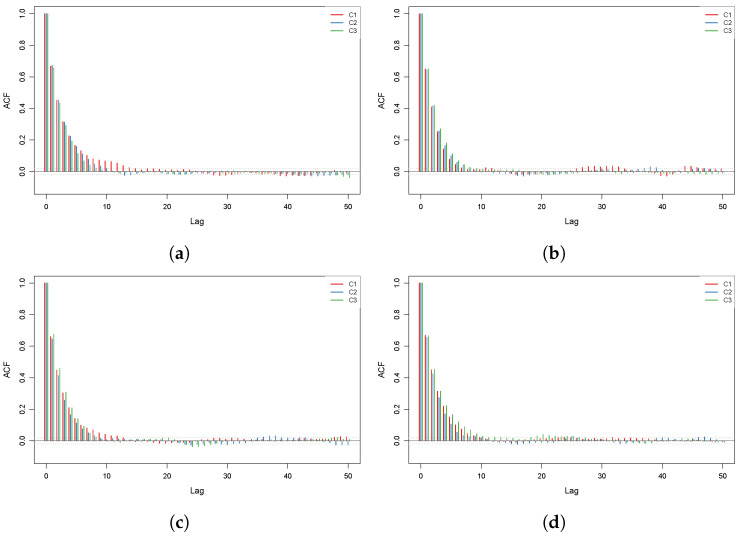
MCMC diagnostics with n=80, m=30, T=(1,1.5). (**a**) ACF of $\beta_{1}$ under informative priors. (**b**) ACF of $\beta_{2}$ under informative priors. (**c**) ACF of $\beta_{1}$ under non-informative priors. (**d**) ACF of $\beta_{2}$ under non-informative priors.

**Figure 5:**
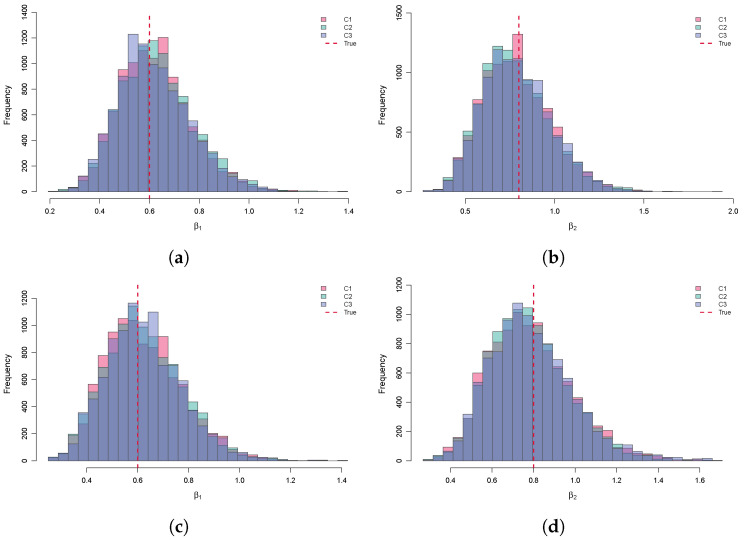
MCMC diagnostics with n=80, m=30, T=(1,1.5). (**a**) Posterior density of $\beta_{1}$ under informative priors. (**b**) Posterior density of $\beta_{2}$ under informative priors. (**c**) Posterior density of $\beta_{1}$ under non-informative priors. (**d**) Posterior density of $\beta_{2}$ under non-informative priors.

To assess the convergence behavior of the MCMC algorithm, the trace plots, autocorrelation plots, and marginal posterior histograms of three parallel chains (C1, C2, C3) under the informative and non-informative priors are displayed in Figure 3, Figure 4 and Figure 5, respectively. The trace plots (Figure 3) indicate that, for each parameter, the three chains mix well and converge rapidly to a common stationary distribution. The autocorrelation plots (Figure 4) show that the autocorrelations decay to zero as the lag increases, indicating adequate mixing and negligible dependence between successive draws. Moreover, the histograms (Figure 5) display an approximately symmetric and unimodal shape centered at the posterior mode, implying that the posterior mean—the Bayes estimator under the squared error loss—provides a reliable point estimate of the underlying parameter.

For each censoring scheme, the performance of the MLEs and Bayesian estimators is assessed in terms of the AB, MSE, IL, and CP. The corresponding results are reported in Table A1, Table A2, Table A3, Table A4, Table A5, Table A6, Table A7, Table A8, Table A9 and Table A10 of Appendix B.

As shown in Table A1, Table A2 and Table A3, the AB and MSE of both the MLE and the Bayesian estimators are close to zero for all parameters. Moreover, for fixed $T_{1}$, the AB and MSE of both estimators tend to decrease as $T_{2}$ increases, and a similar pattern is observed as the effective sample size *m* grows. In most cases, the AB and MSE values produced by the Bayesian estimator under informative priors are slightly smaller than those under non-informative priors, which in turn are slightly smaller than those of the MLE.

As illustrated in Table A4, Table A5, Table A6, Table A7, Table A8, Table A9 and Table A10, the CPs of the ACIs, the HPD credible intervals (under both informative and non-informative priors), and the bootstrap intervals (bootstrap-*t* and bootstrap-*p*) are all close to the nominal confidence level. In most cases, under the same CS, the ILs rank from smallest to largest as follows: bootstrap-*p*, bootstrap-*t*, informative HPD, non-informative HPD, and ACI based on the MLE. As the effective sample size increases, the IL decreases and the CP increases for every method considered.

In summary, the simulation results indicate that the Bayesian point and interval estimators outperform their MLE counterparts in the majority of settings. The Bayesian estimator together with the associated credible interval is therefore the preferred choice whenever prior information on the unknown parameters is available; otherwise, the results based on the non-informative prior can serve as a reliable alternative.

## 6. Real Data Analysis

In this section, we present an analysis of a real-life test dataset to illustrate the inference process presented in [44]. The dataset contains 58 electrodes (segments cut from bars) and was subjected to a high-stress voltage endurance life test. This dataset has also been considered by numerous authors. For example, ref. [13] considered the estimation problems using the competing risk model with T-II PCS Chen distribution. The failures were attributed to one of the following two causes based on an autopsy (Table 2):

- cause 1. Degradation failure: Degradation of the organic material. These failures typically occur later in life.
- cause 2. Early failure: Insulation defects due to processing problems. These failures occur early in life.

Therefore, we obtain 27 failure samples owing to cause 1 and 18 failure samples owing to cause 2. Additionally, there were 13 electrodes still running. To analyze the previous inference progress, we considered only the completely observed samples and left the sample that was still running.

**Figure 6:**
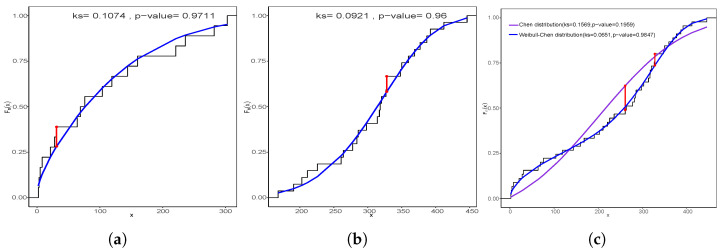
Observation samples versus fitted distribution (The red lines represent the maximum distance between the empirical distribution function and the fitted distribution function). (**a**) The empirical distribution function (black lines) and fitted Chen distribution (blue lines) with failure cause 1. (**b**) The empirical distribution function (black lines) and fitted Weibull distribution (blue lines) with failure cause 2. (**c**) The empirical distribution function (black lines) and fitted Chen–Weibull (blue lines) and Chen distribution (purple lines) with competing risk samples.

**Figure 7:**
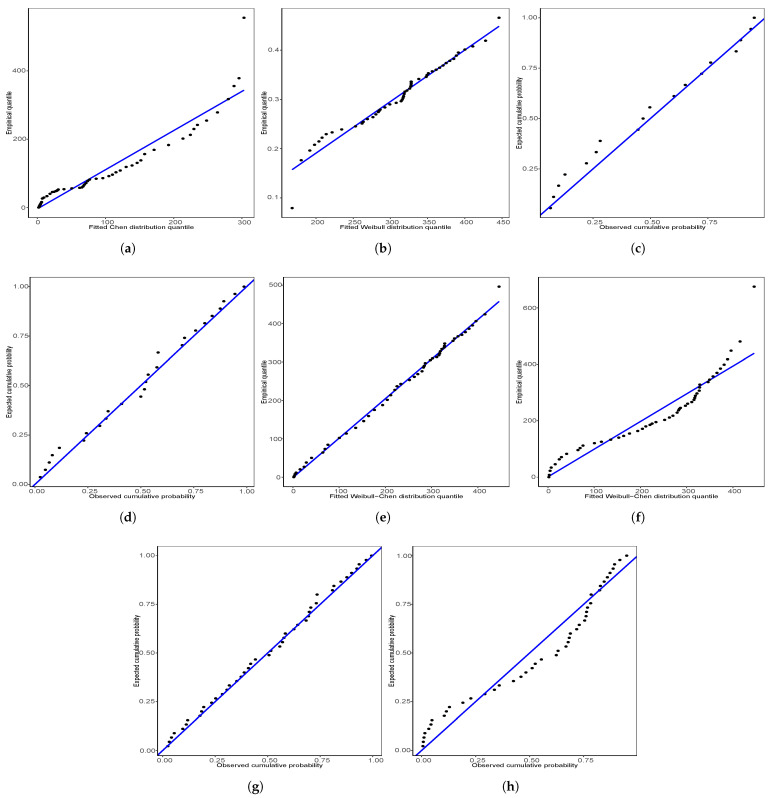
Q–Q and P–P plots of competing risk samples versus fitted distribution. The black dots denote the empirical sample points and the blue line is the reference line y=x, with points close to the line indicating a good fit. (**a**) Q–Q plot of failure cause 1 with Chen distribution. (**b**) Q–Q plot of failure cause 2 with Weibull distribution. (**c**) P–P plot of failure cause 1 with Chen distribution. (**d**) P–P plot of failure cause 2 with Weibull distribution. (**e**) Q–Q plot of competing risk sample with Chen–Weibull distribution. (**f**) Q–Q plot of competing risk sample with Chen-Chen distribution. (**g**) P–P plot of competing risk sample with Chen–Weibull distribution. (**h**) P–P plot of competing risk sample with Chen-Chen distribution.

Before further analysis, we need to determine whether this dataset can be modeled using the C–W distribution. The Kolmogorov–Smirnov (KS) test is an effective method for comparing samples with a reference probability distribution, and thus, we use the KS test to investigate whether the heterogeneous competing risk model, namely C–W competing risk model, is suitable for the dataset. For samples with failure cause 1, we use the Chen distribution to fit the data; the *p*-value of the KS test is 0.9711, and the KS distance is 0.1074. For samples with failure cause 2, we use the Weibull distribution to fit the data; the *p*-value of the KS test is 0.96, and the KS distance is 0.0921. For samples with a complete sample, we use the C–W distribution to fit the data; the *p*-value of the KS test is 0.9847, and the KS distance is 0.0651, The results are reported in Table 3. It can be found that the *p*-values are both relatively large (greater than 0.05). Moreover, in the associated empirical cumulative distribution plots, the KS distances are marked with red dots. The Quantile–Quantile (Q–Q) plots and the Probability–Probability (P–P) plots are shown in Figure 6 and Figure 7, respectively. It can be easily observed that the C–W distribution is a suitable fit for this electrode’s life test dataset. Therefore, we believe that this set of data can be modeled and analyzed using C–W distribution.

**Figure 8:**
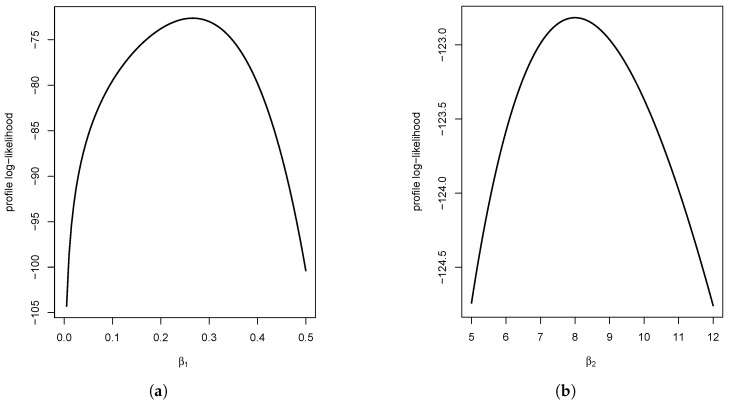
The profile log-likelihood functions. (**a**) Log-likelihood function of $\beta_{1}$. (**b**) Log-likelihood function of $\beta_{2}$.

Based on Table 2, let m=30, $T_{1}=180,$ and $T_{2}=330.$ We obtain IAT-II PCS data using various CSs, as illustrated in Table 4. Figure 8 illustrates that the profile log-likelihood functions of $\beta_{1}$ and $\beta_{2}$ are unimodal using CS S1, which means that the MLE exists and is unique. Additionally, the point and interval estimations of the MLE and Bayesian methods are presented in Table 5, where the results of the MLE resemble those of the Bayes estimate with non-information using S1–S4. It can be observed that the proposed algorithm is suitable and reasonable for processing IAT-II PCS data with heterogeneous competing risks.

## 7. Conclusions

In this study, we develop both frequentist and Bayesian inferential procedures for a heterogeneous competing risks model under the IAT-II PCS, in which heterogeneity is captured through the Chen–Weibull distribution specification. This modeling choice is motivated by the flexibility of the component distributions and their hazard rate functions, which, as demonstrated in our real-data application, yield a noticeably better fit than homogeneous alternatives. For frequentist inference, the Newton–Raphson iteration furnishes the MLEs together with the associated asymptotic and bootstrap confidence intervals; for Bayesian inference, an MCMC sampler under squared-error loss produces posterior estimates and highest posterior density credible intervals. All computations were implemented in R, and the corresponding code is available from the author upon request. Several limitations of the present work suggest avenues for future research. First, only two competing failure causes are considered. While multivariate independent competing risks models can be obtained by a straightforward extension of the bivariate formulation, multivariate dependent competing risks models—particularly those constructed via copulas—call for additional methodological development, as the selection of a high-dimensional copula family and the attendant identifiability issues remain open problems. Second, because the true underlying distribution and dependence structure are seldom known with certainty in practice, model misspecification may inflate estimation bias; semi-parametric and fully nonparametric approaches to competing risks inference therefore warrant further investigation. Third, the proposed methodology could be extended to multi-state component systems under various censoring schemes, so as to capture the performance evolution of components across multiple operating states; recent work in this direction includes that of Shi and Yan [45].

## Figures and Tables

**Table 1:** Censoring schemes using *n* and *m* with $\left(T_{1}=0.8,T_{2}=1.2\right)$, $\left(T_{1}=0.8,T_{2}=1.5\right)$, $(T_{1}=1,$$T_{2}=1.5)$, $\left(T_{1}=0.8,T_{2}=100\right)$, and $\left(T_{1}=100,T_{2}=101\right)$, where $a*k=(\underset{k}{\underset{︸}{a,a,\cdots ,a}})$.

(n,m)		(50,30)	(80,30)	(80,60)
CS	L	(20,0 ∗ 29)	(50,0 ∗ 29)	(20,0 ∗ 59)
	R	(0 ∗ 29,20)	(0 ∗ 29,50)	(0 ∗ 59,20)
	U	(1 ∗ 10,0 ∗ 10,1 ∗ 10)	(2 ∗ 10,1 ∗ 10,2 ∗ 10)	(1 ∗ 10,0 ∗ 40,1 ∗ 10)
	M	(0 ∗ 14,20,0 ∗ 15)	(0 ∗ 14,50,0 ∗ 15)	(0 ∗ 29,20,0 ∗ 30)

**Table 2:** Voltage endurance life test results of 58 electrodes from [44], where D: Degradation failure, E: Early failure and ∘: unfilled electrode.

Hours	Failure Mode	Hours	Failure Mode	Hours	Failure Mode	Hours	Failure Mode	Hours	Failure Mode	Hours	Failure Mode	Hours	Failure Mode
2	E	52	∘	119	E	211	D	282	E	327	D	387	D
3	E	53	∘	135	∘	221	E	284	D	328	D	392	D
5	E	64	E	144	E	226	D	286	D	328	D	412	D
8	E	67	∘	157	∘	236	E	298	D	348	D	446	D
13	∘	69	E	160	E	241	∘	303	E	348	∘		
21	E	76	E	168	D	257	∘	314	D	350	D		
28	E	78	∘	179	∘	261	D	317	D	360	D		
31	E	104	E	191	D	264	D	318	D	369	D		
31	∘	113	∘	203	D	278	D	320	D	377	D		

**Table 3:** Fitting KS test for the data of voltage endurance life test ($a\times 10^{b}=a\left(10^{b}\right)$).

Data	${\hat{\alpha }}_{1}$	${\hat{\beta }}_{1}$	${\hat{\alpha }}_{2}$	${\hat{\beta }}_{2}$	KS	*p*-Value (%)
Cause 1	2.88$\left(10^{-2}\right)$	2.70$\left(10^{-1}\right)$	−	−	1.07$\left(10^{-1}\right)$	97.11
Cause 2	−	−	5.26$\left(10^{0}\right)$	3.37$\left(10^{2}\right)$	9.21 $\left(10^{-2}\right)$	96.00
Complete Data	4.14$\left(10^{-6}\right)$	4.32$\left(10^{-1}\right)$	1.66$\left(10^{-2}\right)$	5.91$\left(10^{-1}\right)$	6.51 $\left(10^{-2}\right)$	98.47

**Table 4:** Improve adaptive Type II progressive censored data with competing risks using four CSs of the real data, where $a*k=(\underset{k}{\underset{︸}{a,a,\cdots ,a}})$.

Scheme 1 (S1): L = (3 ∗ 5,0 ∗ 25), $k_{1}=4,k_{2}=24$									
(2,0)	(21,0)	(69,0)	(144,0)	(203,1)	(211,1)	(221,0)	(226,1)	(236,0)	(261,1)
(264,1)	(278,1)	(282,0)	(284,1)	(286,1)	(298,1)	(303,0)	(314,1)	(317,1)	(318,1)
(320,1)	(327,1)	(328,1)	(328,1)						
Scheme 2 (S2): R = (0 ∗ 25,3 ∗ 5), $k_{1}=12,k_{2}=29$									
(2,0)	(3,0)	(5,0)	(8,0)	(21,0)	(28,0)	(31,0)	(64,0)	(69,0)	(76,0)
(104,0)	(221,0)	(226,1)	(236,0)	(261,1)	(264,1)	(278,1)	(282,0)	(284,1)	(286,1)
(298,1)	(303,0)	(314,1)	(317,1)	(318,1)	(320,1)	(327,1)	(328,1)	(328,1)	
Scheme 3 (S3): M = (0 ∗ 12,3 ∗ 5,0 ∗ 13), $k_{1}=8,k_{2}=27$									
(2,0)	(3,0)	(5,0)	(8,0)	(21,0)	(28,0)	(76,0)	(160,0)	(211,1)	(221,0)
(226,1)	(236,0)	(261,1)	(264,1)	(278,1)	(282,0)	(284,1)	(286,1)	(298,1)	(303,0)
(314,1)	(317,1)	(318,1)	(320,1)	(327,1)	(328,1)	(328,1)			
Scheme 4 (S4): U = (3 ∗ 3,0 ∗ 25,3 ∗ 2), $k_{1}=6,k_{2}=27$									
(2,0)	(21,0)	(69,0)	(144,0)	(160,0)	(168,1)	(191,1)	(203,1)	(211,1)	(221,0)
(226,1)	(236,0)	(261,1)	(264,1)	(278,1)	(282,0)	(284,1)	(286,1)	(298,1)	(303,0)
(314,1)	(317,1)	(318,1)	(320,1)	(327,1)	(328,1)	(328,1)			

**Table 5:** Point and interval estimations of parameters using four CSs ($a\times 10^{b}=a\left(10^{b}\right)$).

	MLE		Bayes	
	**Point**	**ACI**	**Point**	HPD
S1				
$\alpha_{1}$	2.70$\left(10^{-3}\right)$	(3.00$\left(10^{-4}\right)$, 2.81$\left(10^{-2}\right)$)	1.80$\left(10^{-3}\right)$	(6.00$\left(10^{-6}\right)$, 6.90$\left(10^{-3}\right)$)
$\alpha_{2}$	6.64$\left(10^{-21}\right)$	(2.42$\left(10^{-23}\right)$, 1.82$\left(10^{-18}\right)$)	1.32$\left(10^{-21}\right)$	(1.45$\left(10^{-22}\right)$, 4.84$\left(10^{-21}\right)$)
$\beta_{1}$	2.66$\left(10^{-1}\right)$	(1.90$\left(10^{-1}\right)$, 3.72$\left(10^{-1}\right)$)	2.95$\left(10^{-1}\right)$	(2.07$\left(10^{-1}\right)$, 3.57$\left(10^{-1}\right)$)
$\beta_{2}$	7.99$\left(10^{0}\right)$	(7.82$\left(10^{0}\right)$, 8.17$\left(10^{0}\right)$)	8.34$\left(10^{0}\right)$	(8.05$\left(10^{0}\right)$, 8.51$\left(10^{0}\right)$)
S2				
$\alpha_{1}$	1.97$\left(10^{-2}\right)$	(5.20$\left(10^{-3}\right)$, 7.51$\left(10^{-2}\right)$)	1.68$\left(10^{-2}\right)$	(2.00$\left(10^{-3}\right)$, 3.76$\left(10^{-2}\right)$)
$\alpha_{2}$	4.66$\left(10^{-27}\right)$	(2.52$\left(10^{-29}\right)$, 8.62$\left(10^{-25}\right)$)	9.34$\left(10^{-23}\right)$	(1.71$\left(10^{-25}\right)$, 1.42$\left(10^{-23}\right)$)
$\beta_{1}$	1.96$\left(10^{-1}\right)$	(1.37$\left(10^{-1}\right)$, 2.80$\left(10^{-1}\right)$)	2.06$\left(10^{-1}\right)$	(1.46$\left(10^{-1}\right)$, 2.66$\left(10^{-1}\right)$)
$\beta_{2}$	1.04$\left(10^{0}\right)$	(1.02$\left(10^{0}\right)$, 1.06$\left(10^{0}\right)$)	9.35$\left(10^{0}\right)$	(8.97$\left(10^{0}\right)$, 9.55$\left(10^{0}\right)$)
S3				
$\alpha_{1}$	1.53$\left(10^{-2}\right)$	(3.20$\left(10^{-3}\right)$, 7.27$\left(10^{-2}\right)$)	1.17$\left(10^{-2}\right)$	(1.40$\left(10^{-3}\right)$, 2.88$\left(10^{-2}\right)$)
$\alpha_{2}$	1.19$\left(10^{-22}\right)$	(6.63$\left(10^{-25}\right)$, 2.17$\left(10^{-20}\right)$)	8.80$\left(10^{-24}\right)$	(5.74$\left(10^{-24}\right)$, 3.54$\left(10^{-22}\right)$)
$\beta_{1}$	1.96$\left(10^{-1}\right)$	(1.28$\left(10^{-1}\right)$, 2.96$\left(10^{-1}\right)$)	2.12$\left(10^{-1}\right)$	(1.44$\left(10^{-1}\right)$, 2.67$\left(10^{-1}\right)$)
$\beta_{2}$	8.60$\left(10^{0}\right)$	(8.42$\left(10^{0}\right)$, 8.80$\left(10^{0}\right)$)	8.77$\left(10^{0}\right)$	(8.41$\left(10^{0}\right)$, 8.99$\left(10^{0}\right)$)
S4				
$\alpha_{1}$	2.70$\left(10^{-3}\right)$	(3.00$\left(10^{-4}\right)$, 2.58$\left(10^{-2}\right)$)	1.66$\left(10^{-2}\right)$	(2.00$\left(10^{-3}\right)$, 3.60$\left(10^{-2}\right)$)
$\alpha_{2}$	4.15$\left(10^{-16}\right)$	(1.54$\left(10^{-18}\right)$, 1.12$\left(10^{-13}\right)$)	2.64$\left(10^{-24}\right)$	(2.11$\left(10^{-25}\right)$, 1.02$\left(10^{-23}\right)$)
$\beta_{1}$	2.70$\left(10^{-1}\right)$	(1.98$\left(10^{-1}\right)$, 3.68$\left(10^{-1}\right)$)	2.07$\left(10^{-1}\right)$	(1.52$\left(10^{-1}\right)$, 2.66$\left(10^{-1}\right)$)
$\beta_{2}$	6.10$\left(10^{0}\right)$	(5.94$\left(10^{0}\right)$, 6.27$\left(10^{0}\right)$)	9.35$\left(10^{0}\right)$	(9.03$\left(10^{0}\right)$, 9.54$\left(10^{0}\right)$)

**Figure A1:**
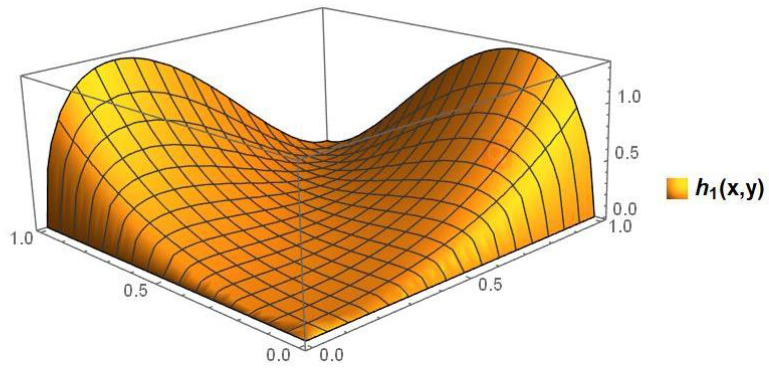
Plot of $h_{1}\left(x,y\right)$.

**Figure A2:**
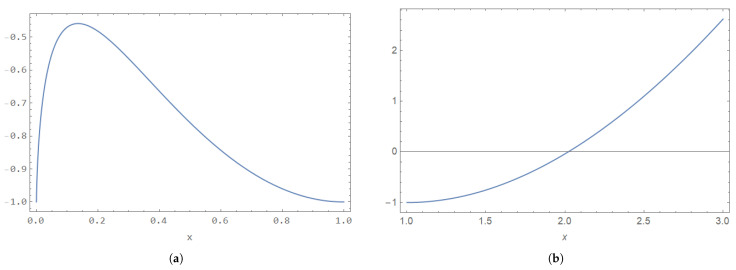
Plot of $h_{3}\left(x\right)$. (**a**) For 0≤x≤1. (**b**) For x>1.

## Data Availability

The original contributions presented in this study are included in the article. Further inquiries can be directed to the corresponding author.

## Abbreviations

The following abbreviations are used in this manuscript:

MCMC	Markov Chain Monte Carlo
HPD	Highest Posterior Density
CDF	Cumulative Distribution Function
PDF	Probability Density Function
MLE	Maximum Likelihood Estimate
C–W distribution	Chen–Weibull distribution
*n*	total number of test units
$n_{1}$	total number of observed failures during the experiment
$n_{2}$	number of failures observed before the first threshold $T_{1}$
$T_{1}$	first pre-specified time threshold ($0<T_{1}<T_{2}$)
$T_{2}$	second pre-specified time threshold (maximum allowable duration)
$R_{i}$	pre-specified number of units removed at the *i*th failure
$t^{*}$	actual termination time, $t^{*}=\min\left\{X_{m:m:n},T_{2}\right\}$
$R^{*}$	final number of removed units at the termination time
$\delta^{*}$	indicator of the failure cause at the termination time, $\delta^{*}=\delta_{n_{1}}\cdot I\left(t^{*}=X_{m:m:n}\right)$

## Appendix A. Proof of Theorem 1

Before proceeding to the main results, we first present a lemma that is very useful in the sequel. First, suppose that functions $a_{i}\left(x\right)$ and $c_{i}\left(x\right)\;\left(i=1,2,\ldots ,n\right)$ are defined on (0,∞), and let $\left\{b_{i},i=1,2,\ldots ,n\right\}$ be a constant sequence.

**Lemma** **A1.** *If $\lim_{x\to 0}a_{i}\left(x\right)=0,\lim_{x\to 0}c_{i}\left(x\right)=0,$ and $\lim_{x\to 0}\frac{a_{i}\left(x\right)}{c_{i}\left(x\right)}=1$, then*

(A1) $$\lim_{x\to 0}\frac{\sum_{i=1}^{n}a_{i}\left(x\right)b_{i}}{\sum_{i=1}^{n}c_{i}\left(x\right)}=\lim_{x\to 0}\sum_{j=1}^{n}\left(\frac{\sum_{i=j}^{n}a_{i}\left(x\right)}{\sum_{i=1}^{n}c_{i}\left(x\right)}\right)\left(b_{j}-b_{j-1}\right)=b_{n},$$

*where $b_{0}=0$*.

For convenience, let

(A2) $$\frac{\eta_{1}^{\prime }\left(\beta_{1}\right)}{\eta_{1}\left(\beta_{1}\right)}=\frac{\sum_{i=1}^{n_{1}}{x_{i}}^{\beta_{1}}e^{{x_{i}}^{\beta_{1}}}\log x_{i}+\sum_{i=1}^{n_{2}}R_{i}{x_{i}}^{\beta_{1}}e^{{x_{i}}^{\beta_{1}}}\log x_{i}+R^{*}{t^{*}}^{\beta_{1}}e^{{t^{*}}^{\beta_{1}}}\log t^{*}}{\sum_{i=1}^{n_{1}}\left(e^{{x_{i}}^{\beta_{1}}}-1\right)+\sum_{i=1}^{n_{2}}R_{i}\left(e^{{x_{i}}^{\beta_{1}}}-1\right)+R^{*}\left(e^{{t^{*}}^{\beta_{1}}}-1\right)}=\frac{\sum_{i=1}^{n_{1}+R^{*}}d_{i}{t_{i}}^{\beta_{1}}e^{{t_{i}}^{\beta_{1}}}\log t_{i}}{\sum_{i=1}^{n_{1}+R^{*}}d_{i}\left(e^{{t_{i}}^{\beta_{1}}}-1\right)},$$

where

$$d_{i}=\left\{\begin{array}{cc} R_{i}+1, & i=1,2,\ldots ,n_{2},\\ 1, & i=n_{2}+1,n_{2}+2,\ldots ,n_{1}+R^{*} \end{array}\right.$$

and

$$t_{i}=\left\{\begin{array}{cc} x_{i}, & i=1,2,\ldots ,n_{1},\\ t^{*}, & i=n_{1}+1,n_{1}+2,\ldots ,n_{1}+R^{*}. \end{array}\right.$$

From (14), we have $\lim_{\beta_{1}\to 0}\partial l_{1}\left(\beta_{1}\right)/\partial \beta_{1}=\infty ,$ without loss of generality, assume that $\lim_{\beta_{1}\to \infty }\partial l_{1}\left(\beta_{1}\right)/\partial \beta_{1}$ exists. Next, we prove that $\lim_{\beta_{1}\to \infty }\partial l_{1}\left(\beta_{1}\right)/\partial \beta_{1}<0$ in the following three cases:

Case 1: For $0<t_{i}<1\left(i=1,2,\ldots ,n_{1}+R^{*}\right).$ We have

$$\lim_{\beta_{1}\to \infty }t_{i}^{\beta_{1}}=0⟹t_{i}^{\beta_{1}}e^{t_{i}^{\beta_{1}}}∼e^{t_{i}^{\beta_{1}}}-1,$$

in (A2), let

(A3) $$\begin{array}{cc} a_{i}\left(t_{i}^{\beta_{1}}\right) & =d_{i}{t_{i}}^{\beta_{1}}e^{{t_{i}}^{\beta_{1}}},\;\;b_{i}=\log t_{i},\;\;c_{i}\left(t_{i}^{\beta_{1}}\right)=d_{i}\left(e^{t_{i}^{\beta_{1}}}-1\right), \end{array}$$

then, combine with Lemma A1, we have

(A4) $$\begin{array}{cc} \lim_{\beta_{1}\to \infty }\frac{\eta_{1}^{\prime }\left(\beta_{1}\right)}{\eta_{1}\left(\beta_{1}\right)} & =\log t^{*}. \end{array}$$

Therefore

(A5) $$\begin{array}{cc} \lim_{\beta_{1}\to \infty }\frac{\partial l_{1}\left(\beta_{1}\right)}{\partial \beta_{1}} & =-m_{1}\log t^{*}+\sum_{i=1}^{n_{1}}I\left(\delta_{i}=1\right)\log x_{i}<0. \end{array}$$

Case 2: For $t_{i}\geq 1\left(i=1,2,\ldots ,n_{1}+R^{*}\right).$ Note that,

(A6) $$\begin{array}{c} \frac{t_{i}^{\beta_{1}}e^{t_{i}^{\beta_{1}}}}{\left(t_{i}^{\beta_{1}}+1\right)}-\left(e^{t_{i}^{\beta_{1}}}-1\right)<0⟹\frac{\sum_{i=1}^{n_{1}+R^{*}}d_{i}\frac{t_{i}^{\beta_{1}}e^{t_{i}^{\beta_{1}}}}{t_{i}^{\beta_{1}}+1}}{\sum_{i=1}^{n_{1}+R^{*}}d_{i}\left(e^{t_{i}^{\beta_{1}}}-1\right)}<1, \end{array}$$

thus, according to (A2), we have

(A7) $$\begin{array}{cc} \frac{\eta_{1}^{\prime }\left(\beta_{1}\right)}{\eta_{1}\left(\beta_{1}\right)} & =\frac{\sum_{i=1}^{n_{1}+R^{*}}d_{i}\frac{t_{i}^{\beta_{1}}e^{t_{i}^{\beta_{1}}}}{t_{i}^{\beta_{1}}+1}\left(t_{i}^{\beta_{1}}+1\right)\log t_{i}}{\sum_{i=1}^{n_{1}+R^{*}}d_{i}\left(e^{t_{i}^{\beta_{1}}}-1\right)}<\left(t_{i}^{*\beta_{1}}+1\right)\log t_{i}^{*}. \end{array}$$

Therefore, for (14)

(A8) $$\begin{array}{cc} \frac{\partial l_{1}\left(\beta_{1}\right)}{\partial \beta_{1}}-\frac{m_{1}}{\beta_{1}} & <-m_{1}\left(t_{i}^{*\beta_{1}}+1\right)\log t_{i}^{*}+\frac{m_{1}}{\beta_{1}}+\sum_{i=1}^{n_{1}}\left(x_{i}^{\beta_{1}}+1\right)I\left(\delta_{i}=1\right)\log x_{i}\\ \; & =\sum_{i=1}^{n_{1}}\left[\left(x_{i}^{\beta_{1}}+1\right)\log x_{i}-\left(t_{i}^{*\beta_{1}}+1\right)\log t_{i}^{*}\right]I\left(\delta_{i}=1\right)\\ \; & <0. \end{array}$$

By the number-preserving nature of the limit,

(A9) $$\begin{array}{cc} \lim_{\beta_{1}\to \infty }\frac{\partial l_{1}\left(\beta_{1}\right)}{\partial \beta_{1}} & <0. \end{array}$$

Case 3: For some $0<t_{i}<1,$ and other $t_{i}\geq 1.$ Without loss of generality, assume that $0<t_{1}<1,$ and $t_{i}\geq 1\left(i=2,3,\ldots ,n_{1}+R^{*}\right).$ In particular, let i=1 in Case 1 and let $i=2,3,\ldots ,n_{1}+R^{*}$ in Case 2, which further leads to

(A10) $$\begin{array}{cc} \lim_{\beta_{1}\to \infty }\frac{\partial l_{1}\left(\beta_{1}\right)}{\partial \beta_{1}} & <0. \end{array}$$

Hence, the root of likelihood Equation (14) exists. In what following, it is shown the MLE of $\beta_{1}$ is unique.

Now, to check $\partial^{2}l_{1}\left(\beta_{1}\right)/\partial \beta_{1}^{2}<0$. According to (12), we have

(A11) $$\begin{array}{cc} \frac{\partial^{2}l_{1}\left(\beta_{1}\right)}{\partial \beta_{1}^{2}} & =-m_{1}\left(\frac{\eta_{1}\left(\beta_{1}\right)\eta_{1}^{\prime \prime }\left(\beta_{1}\right)-{\left(\eta_{1}^{\prime }\left(\beta_{1}\right)\right)}^{2}}{{\left(\eta_{1}\left(\beta_{1}\right)\right)}^{2}}\right)+\sum_{i=1}^{n_{1}}\left(x_{i}^{\beta_{1}}{\left(\log x_{i}\right)}^{2}-\frac{1}{\beta_{1}^{2}}\right)I\left(\delta_{i}=1\right), \end{array}$$

Next, just verify

(A12) $$\begin{array}{cc} \frac{\eta_{1}\left(\beta_{1}\right)\eta_{1}^{\prime \prime }\left(\beta_{1}\right)-{\left(\eta_{1}^{\prime }\left(\beta_{1}\right)\right)}^{2}}{{\left(\eta_{1}\left(\beta_{1}\right)\right)}^{2}} & \overset{sgn}{=}\eta_{1}\left(\beta_{1}\right)\eta_{1}^{\prime \prime }\left(\beta_{1}\right)-{\left(\eta_{1}^{\prime }\left(\beta_{1}\right)\right)}^{2}\geq 0, \end{array}$$

and

(A13) $$\begin{array}{cc} x_{i}^{\beta_{1}}{\left(\log x_{i}\right)}^{2}-\frac{1}{\beta_{1}^{2}} & \leq 0. \end{array}$$

For (21) and (A12), we have

$$\begin{array}{cc} \; & \eta_{1}\left(\beta_{1}\right)\eta_{1}^{\prime \prime }\left(\beta_{1}\right)-{\left(\eta_{1}^{\prime }\left(\beta_{1}\right)\right)}^{2}\\ = & \left(\sum_{1\leq i\leq n_{1}}\left(e^{x_{i}^{\beta_{1}}}-1\right)+\sum_{1\leq i\leq n_{2}}R_{i}\left(e^{x_{i}^{\beta_{1}}}-1\right)+R^{*}\left(e^{t^{*\beta_{1}}}-1\right)\right)(\sum_{1\leq i\leq n_{1}}x_{i}^{\beta_{1}}\left(x_{i}^{\beta_{1}}+1\right)e^{x_{i}^{\beta_{1}}}{\left(\log x_{i}\right)}^{2}\\ \; & +\sum_{1\leq i\leq n_{2}}R_{i}x_{i}^{\beta_{1}}\left(x_{i}^{\beta_{1}}+1\right)e^{x_{i}^{\beta_{1}}}{\left(\log x_{i}\right)}^{2}+R^{*}t^{*\beta_{1}}\left(t^{*\beta_{1}}+1\right)e^{t^{*\beta_{1}}}{\left(\log t^{*}\right)}^{2})-(\sum_{1\leq i\leq n_{1}}x_{i}^{\beta_{1}}e^{x_{i}^{\beta_{1}}}\log x_{i}\\ \; & +\sum_{1\leq i\leq n_{2}}R_{i}x_{i}^{\beta_{1}}e^{x_{i}^{\beta_{1}}}\log x_{i}+R^{*}t^{*\beta_{1}}e^{t^{*\beta_{1}}}\log t^{*}{)}^{2} \end{array}$$

(A14) $$\begin{array}{cc} \overset{sgn}{=} & \sum_{1\leq i<j\leq n_{1}}h_{1}\left(x_{i}^{\beta_{1}},x_{j}^{\beta_{1}}\right)+\sum_{1\leq i<j\leq n_{2}}R_{i}R_{j}h_{1}\left(x_{i}^{\beta_{1}},x_{j}^{\beta_{1}}\right)+\sum_{1\leq i\leq n_{1},1\leq j\leq n_{2}}R_{i}h_{1}\left(x_{i}^{\beta_{1}},x_{j}^{\beta_{1}}\right)\\ \; & +\sum_{1\leq i\leq n_{1}}R^{*}h_{1}\left(x_{i}^{\beta_{1}},t^{*\beta_{1}}\right)+\sum_{1\leq j\leq n_{2}}R^{*}h_{1}\left(t^{*\beta_{1}},x_{i}^{\beta_{1}}\right)+\sum_{1\leq i\leq n_{1}}h_{2}\left(x_{i}^{\beta_{1}}\right)+R^{*2}h_{2}\left(t^{*\beta_{1}}\right)\\ \; & +\sum_{1\leq j\leq n_{2}}R_{i}^{2}h_{2}\left(x_{i}^{\beta_{1}}\right)\\ \geq & 0, \end{array}$$

where

(A15) $$\begin{array}{cc} h_{1}\left(x,y\right) & =x\left(1+x\right)e^{x}\left(e^{y}-1\right){\left(\log x\right)}^{2}+y\left(1+y\right)e^{y}\left(e^{x}-1\right){\left(\log y\right)}^{2}\\ \; & \;-2xye^{x+y}\log x\log y,\;\;0<x,y\leq 1, \end{array}$$

and

(A16) $$\begin{array}{cc} h_{2}\left(x\right) & =xe^{x}\left(e^{x}-x-1\right){\left(\log x\right)}^{2}\geq 0,\;\;0<x\leq 1. \end{array}$$

For (A14), it should be noted that it is difficult to analyze $h_{1}\left(x,y\right)$ directly because of the complexity of its analytic equation, so the global minimum of $h_{1}\left(x,y\right)$ can be obtained with the help of the NMinimize function in Mathematical (Version 14.3) software, as well as the minimum value point, and the 3D plot can be obtained using function Plot3D, that is,

$$\left\{\begin{array}{c} x=2.288\times 10^{-6},\\ y=2.289\times 10^{-6}, \end{array}\right.⟹h_{1}{\left(x,y\right)}_{min}=2.022\times 10^{-15}>0.$$

Therefore, for all 0<x,y<1, $h_{1}\left(x,y\right)>0$ always holds, which is further verified in Figure A1.

In addition, for (A13),

(A17) $$\begin{array}{cc} x_{i}^{\beta_{1}}{\left(\log x_{i}\right)}^{2}-\frac{1}{\beta_{1}^{2}} & \overset{sgn}{=}x_{i}^{\beta_{1}}{\left(\log x_{i}\right)}^{2}\beta_{1}^{2}-1=x_{i}^{\beta_{1}}{\left(\log x_{i}^{\beta_{1}}\right)}^{2}-1:=h_{3}\left(x_{i}^{\beta_{1}}\right). \end{array}$$

It is observed that, for any $\beta_{1}\geq 0$ and 0<x≤1, $h_{3}\left(x\right)<0$ always holds (see Figure A2a), however, from Figure A2b, it is clear that for x>1, $h_{3}\left(x\right)<0$ doses not always hold. Therefore, for any $\beta_{1}>0,$ and $0<x_{i}\leq 1\left(i=1,2,\ldots ,n\right),$ (A13) holds.

In summary, we verify of $\frac{\partial^{2}l_{1}\left(\beta_{1}\right)}{\partial \beta_{1}^{2}}<0$, combine with $\left\{\begin{array}{c} \lim_{\beta_{1}\to 0}\frac{\partial l_{1}\left(\beta_{1}\right)}{\partial \beta_{1}}=\infty ,\\ \lim_{\beta_{1}\to \infty }\frac{\partial l_{1}\left(\beta_{1}\right)}{\partial \beta_{1}}<0, \end{array}\right.$ and thus, $l_{1}\left(\beta_{1}\right)$ is a unimodal function. This ends the proof of Theorem 1.

## Appendix B. The Relevant Tables of Simulation

**Table A1:** ABs and MSEs (within bracket) for MLE and Bayes point estimation of parameters under ($T_{1}=0.8,T_{2}=1.2$) and ($T_{1}=0.8,T_{2}=1.5$).

(n,m)	CS	$T_{1}=0.8,T_{2}=1.2$				$T_{1}=0.8,T_{2}=1.5$			
		${\hat{\alpha }}_{1}$	${\hat{\alpha }}_{2}$	${\hat{\beta }}_{1}$	${\hat{\beta }}_{2}$	${\hat{\alpha }}_{1}$	${\hat{\alpha }}_{2}$	${\hat{\beta }}_{1}$	${\hat{\beta }}_{2}$
MLE									
(50,30)	L	0.0224 (0.0076)	0.0312 (0.0245)	0.1299 (0.0977)	0.0718 (0.1046)	0.0094 (0.0058)	0.0297 (0.0208)	0.0943 (0.0623)	0.0704 (0.0964)
	R	0.0143 (0.0047)	0.0186 (0.0127)	0.0820 (0.0544)	0.0747 (0.0681)	0.0108 (0.0049)	0.0164 (0.0125)	0.0679 (0.0446)	0.0674 (0.0597)
	U	0.0174 (0.0055)	0.0307 (0.0173)	0.0986 (0.0630)	0.0796 (0.0739)	0.0086 (0.0043)	0.0271 (0.0150)	0.0674 (0.0419)	0.0839 (0.0657)
	M	0.0393 (0.0102)	0.0479 (0.0253)	0.1316 (0.0871)	0.1019 (0.0937)	0.0240 (0.0077)	0.0397 (0.0201)	0.0998 (0.0591)	0.0924 (0.0813)
(80,30)	L	0.0208 (0.0076)	−0.0018 (0.0331)	0.1129 (0.0808)	−0.0961 (0.1757)	0.0091 (0.0059)	−0.0008 (0.0307)	0.0784 (0.0567)	−0.0931 (0.1680)
	R	0.0477 (0.0148)	0.0225 (0.0226)	0.1194 (0.0735)	0.0396 (0.0594)	0.0485 (0.0161)	0.0192 (0.0210)	0.1197 (0.0751)	0.0361 (0.0565)
	U	0.0289 (0.0068)	0.0310 (0.0165)	0.0942 (0.0475)	0.0498 (0.0519)	0.0189 (0.0056)	0.0243 (0.0142)	0.0711 (0.0369)	0.0461 (0.0459)
	M	0.0513 (0.0259)	0.0541 (0.0333)	0.1187 (0.0642)	0.0534 (0.0681)	0.0321 (0.0088)	0.0440 (0.0258)	0.0823 (0.0432)	0.0422 (0.0654)
(80,60)	L	0.0094 (0.0029)	0.0182 (0.0099)	0.0596 (0.0331)	0.0486 (0.0465)	0.0023 (0.0024)	0.0179 (0.0087)	0.0367 (0.0223)	0.0395 (0.0423)
	R	0.0066 (0.0020)	0.0114 (0.0069)	0.0430 (0.0228)	0.0388 (0.0334)	0.0007 (0.0018)	0.0134 (0.0062)	0.0203 (0.0156)	0.0423 (0.0299)
	U	0.0086 (0.0024)	0.0129 (0.0081)	0.0521 (0.0269)	0.0386 (0.0372)	0.0016 (0.0020)	0.0173 (0.0076)	0.0289 (0.0176)	0.0496 (0.0340)
	M	0.0142 (0.0030)	0.0154 (0.0091)	0.0624 (0.0297)	0.0418 (0.0396)	0.0076 (0.0023)	0.0160 (0.0079)	0.0407 (0.0240)	0.0492 (0.0362)
Information Bayes									
(50,30)	L	−0.0248 (0.0054)	−0.0359 (0.0175)	0.0533 (0.0487)	0.0525 (0.0713)	−0.0283 (0.0052)	−0.0347 (0.0161)	0.0456 (0.0425)	0.0633 (0.0777)
	R	−0.0079 (0.0038)	−0.0178 (0.0109)	0.0479 (0.0368)	0.0452 (0.0546)	−0.0074 (0.0036)	−0.0181 (0.0102)	0.0478 (0.0331)	0.0550 (0.0561)
	U	−0.0164 (0.0041)	0.0311 (0.0130)	0.0370 (0.0354)	0.0264 (0.0541)	−0.0179 (0.0038)	−0.0210 (0.0121)	0.0269 (0.0291)	0.0551 (0.0534)
	M	−0.0137 (0.0044)	−0.0322 (0.0144)	0.0295 (0.0331)	0.0108 (0.0535)	−0.0170 (0.0039)	−0.0289 (0.0141)	0.0180 (0.0291)	0.0276 (0.0512)
(80,30)	L	−0.0240 (0.0053)	−0.0360 (0.0175)	0.0622 (0.0495)	0.0293 (0.0673)	−0.0255 (0.0049)	−0.0335 (0.0152)	0.0421 (0.0393)	0.0544 (0.0714)
	R	0.0164 (0.0069)	−0.0220 (0.0132)	0.0668 (0.0403)	−0.0102 (0.0379)	0.0169 (0.0071)	−0.0186 (0.0135)	0.0673 (0.0432)	−0.0042 (0.0385)
	U	−0.0078 (0.0040)	−0.0212 (0.0119)	0.0362 (0.0286)	−0.0011 (0.0367)	−0.0098 (0.0038)	−0.0162 (0.0108)	0.0341 (0.0268)	0.0164 (0.0382)
	M	−0.0120 (0.0052)	−0.0384 (0.0160)	0.0155 (0.0297)	−0.0290 (0.0419)	−0.0184 (0.0042)	−0.0247 (0.0144)	−0.0040 (0.0218)	−0.0114 (0.0378)
(80,60)	L	−0.0123 (0.0026)	−0.0193 (0.0087)	0.0296 (0.0247)	0.0278 (0.0368)	−0.0152 (0.0024)	−0.0138 (0.0075)	0.0154 (0.0168)	0.0218 (0.0340)
	R	−0.0115 (0.0020)	−0.0152 (0.0065)	0.0167 (0.0182)	0.0181 (0.0290)	−0.0114 (0.0017)	−0.0089 (0.0059)	0.0058 (0.0138)	0.0321 (0.0275)
	U	−0.0117 (0.0022)	−0.0161 (0.0072)	0.0255 (0.0222)	0.0144 (0.0306)	−0.0111 (0.0020)	−0.0130 (0.0066)	0.0094 (0.0145)	0.0379 (0.0294)
	M	−0.0080 (0.0022)	−0.0136 (0.0073)	0.0243 (0.0209)	0.0114 (0.0306)	−0.0104 (0.0021)	−0.0103 (0.0072)	0.0101 (0.0155)	0.0242 (0.0294)
Non-information Bayes									
(50,30)	L	−0.0237 (0.0057)	−0.0376 (0.0197)	0.0843 (0.6955)	0.0579 (0.1058)	−0.0290 (0.0054)	−0.0292 (0.0165)	0.0551 (0.1458)	0.0588 (0.0862)
	R	−0.0092 (0.0042)	−0.0240 (0.0120)	0.0541 (0.0453)	0.0340 (0.0559)	−0.0082 (0.0041)	−0.0138 (0.0110)	0.0638 (0.0448)	0.0616 (0.0596)
	U	−0.0137 (0.0048)	−0.0268 (0.0166)	0.0372 (0.0435)	0.0258 (0.0637)	−0.0183 (0.0046)	−0.0290 (0.0149)	0.0171 (0.0320)	0.0305 (0.0630)
	M	−0.0185 (0.0041)	−0.0273 (0.0142)	0.0391 (0.0420)	0.0414 (0.0649)	−0.0169 (0.0039)	−0.0164 (0.0121)	0.0335 (0.0316)	0.0451 (0.0561)
(80,30)	L	−0.0238 (0.0057)	−0.0351 (0.0194)	0.0636 (0.0653)	0.0483 (0.1039)	−0.0284 (0.0051)	−0.0282 (0.0179)	0.0500 (0.0419)	0.0608 (0.0868)
	R	0.0204 (0.0085)	−0.0171 (0.0172)	0.0817 (0.0555)	0.0037 (0.0523)	0.0219 (0.0095)	−0.0188 (0.0164)	0.0756 (0.0531)	−0.0073 (0.0488)
	U	−0.0067 (0.0044)	−0.0214 (0.0141)	0.0340 (0.0324)	0.0026 (0.0435)	−0.0061 (0.0042)	−0.0174 (0.0117)	0.0447 (0.0342)	0.0151 (0.0385)
	M	−0.0131 (0.0054)	−0.0363 (0.0187)	0.0116 (0.0287)	−0.0276 (0.0476)	−0.0194 (0.0046)	−0.0322 (0.0174)	−0.00004 (0.0246)	−0.0252 (0.0415)
(80,60)	L	−0.0126 (0.0028)	−0.0244 (0.0090)	0.0311 (0.0256)	0.0186 (0.0391)	−0.0147 (0.0025)	−0.0092 (0.0082)	0.0227 (0.0189)	0.0310 (0.0382)
	R	−0.0100 (0.0019)	−0.0161 (0.0066)	0.0169 (0.0191)	0.0220 (0.0315)	−0.0107 (0.0018)	−0.0087 (0.0061)	0.0067 (0.0144)	0.0278 (0.0285)
	U	−0.0109 (0.0022)	−0.0128 (0.0076)	0.0287 (0.0214)	0.0211 (0.0357)	−0.0143 (0.0020)	−0.0099 (0.0065)	0.0102 (0.0165)	0.0343 (0.0309)
	M	−0.0064 (0.0024)	−0.0169 (0.0074)	0.0240 (0.0225)	0.0061 (0.0307)	−0.0102 (0.0020)	−0.0096 (0.0073)	0.0127 (0.0161)	0.0237 (0.0302)

**Table A2:** ABs and MSEs (within bracket) for MLE and Bayes point estimation of parameters under ($T_{1}=1,T_{2}=1.5$) and ($T_{1}=0.8,T_{2}=100$).

(n,m)	CS	$T_{1}=1,T_{2}=1.5$				$T_{1}=0.8,T_{2}=100$			
		${\hat{\alpha }}_{1}$	${\hat{\alpha }}_{2}$	${\hat{\beta }}_{1}$	${\hat{\beta }}_{2}$	${\hat{\alpha }}_{1}$	${\hat{\alpha }}_{2}$	${\hat{\beta }}_{1}$	${\hat{\beta }}_{2}$
MLE									
(50,30)	L	0.0084 (0.0056)	0.0304 (0.0216)	0.0924 (0.0640)	0.0713 (0.0946)	−0.0081 (0.0037)	0.0293 (0.0204)	−0.0308 (0.0151)	0.0425 (0.1175)
	R	0.0088 (0.0043)	0.0167 (0.0124)	0.0613 (0.0422)	0.0652 (0.0609)	0.0077 (0.0043)	0.0164 (0.0118)	0.0613 (0.0423)	0.0702 (0.0627)
	U	0.0108 (0.0048)	0.0308 (0.0159)	0.0728 (0.0434)	0.0864 (0.0665)	−0.0002 (0.0036)	0.0250 (0.0130)	0.0259 (0.0251)	0.0775 (0.0521)
	M	0.0232 (0.0070)	0.0399 (0.0211)	0.0992 (0.0594)	0.0948 (0.0811)	−0.0053 (0.0032)	0.0399 (0.0166)	−0.0228 (0.0158)	0.0877 (0.0756)
(80,30)	L	0.0098 (0.0060)	−0.0046 (0.0303)	0.0792 (0.0572)	−0.0920 (0.1710)	−0.0121 (0.0040)	−0.0132 (0.0360)	−0.0335 (0.0188)	−0.1730 (0.2527)
	R	0.0486 (0.0211)	0.0174 (0.0208)	0.1230 (0.0781)	0.0322 (0.0577)	0.0478 (0.0152)	0.0205 (0.0251)	0.1190 (0.0727)	0.0353 (0.0586)
	U	0.0201 (0.0053)	0.0250 (0.0144)	0.0770 (0.0402)	0.0484 (0.0466)	0.0144 (0.0052)	0.0194 (0.0137)	0.0548 (0.0332)	0.0372 (0.0439)
	M	0.0310 (0.0084)	0.0457 (0.0261)	0.0845 (0.0451)	0.0436 (0.0659)	−0.0069 (0.0034)	0.0283 (0.0228)	−0.0266 (0.0205)	−0.0327 (0.1533)
(80,60)	L	0.0030 (0.0024)	0.0177 (0.0087)	0.0353 (0.0220)	0.0530 (0.0412)	−0.0068 (0.0019)	0.0342 (0.0091)	−0.0405 (0.0067)	0.1194 (0.0385)
	R	0.0014 (0.0017)	0.0135 (0.0066)	0.0233 (0.0157)	0.0469 (0.0299)	−0.0036 (0.0016)	0.0174 (0.0058)	−0.0040 (0.0107)	0.0533 (0.0264)
	U	0.0006 (0.0019)	0.0172 (0.0075)	0.0290 (0.0183)	0.0471 (0.0327)	−0.0068 (0.0016)	0.0243 (0.0067)	−0.0192 (0.0085)	0.0743 (0.0285)
	M	0.0078 (0.0023)	0.0185 (0.0085)	0.0427 (0.0212)	0.0465 (0.0346)	−0.0060 (0.0016)	0.0321 (0.0077)	−0.0353 (0.0073)	0.1044 (0.0325)
Information Bayes									
(50,30)	L	−0.0277 (0.0050)	−0.0266 (0.0156)	0.0472 (0.0416)	0.0543 (0.0717)	−0.0240 (0.0037)	0.0043 (0.0136)	−0.0241 (0.0115)	0.1493 (0.0711)
	R	−0.0087 (0.0036)	−0.0106 (0.0107)	0.0446 (0.0337)	0.0565 (0.0493)	−0.0067 (0.0037)	−0.0127 (0.0114)	0.0412 (0.0333)	0.0676 (0.0578)
	U	−0.0173 (0.0039)	−0.0178 (0.0117)	0.0273 (0.0273)	0.0403 (0.0514)	−0.0207 (0.0035)	−0.0043 (0.0107)	0.0267 (0.0242)	0.0797 (0.0488)
	M	−0.0199 (0.0040)	−0.0263 (0.0141)	0.0172 (0.0288)	0.0287 (0.0543)	−0.0214 (0.0033)	0.0067 (0.0116)	−0.0259 (0.0124)	0.1133 (0.0514)
(80,30)	L	−0.0284 (0.0049)	−0.0288 (0.0163)	0.0443 (0.0353)	0.0486 (0.0669)	−0.0298 (0.0040)	0.0098 (0.0135)	−0.0128 (0.0109)	0.1356 (0.0695)
	R	0.0159 (0.0063)	−0.0232 (0.0132)	0.0687 (0.0417)	0.0027 (0.0429)	0.0169 (0.0071)	−0.0168 (0.0135)	0.0712 (0.0431)	−0.0001 (0.0432)
	U	−0.0078 (0.0040)	−0.0188 (0.0120)	0.0333 (0.0263)	0.0067 (0.0369)	−0.0051 (0.0043)	−0.0102 (0.0104)	0.0407 (0.0279)	0.0257 (0.0397)
	M	−0.0166 (0.0045)	−0.0282 (0.0146)	0.0056 (0.0221)	−0.0122 (0.0383)	−0.0216 (0.0035)	0.0129 (0.0119)	−0.0189 (0.0118)	0.0635 (0.0336)
(80,60)	L	−0.0142 (0.0023)	−0.0133 (0.0082)	0.0161 (0.0185)	0.0337 (0.0373)	−0.0179 (0.0019)	0.0185 (0.0080)	−0.0362 (0.0066)	0.1245 (0.0409)
	R	−0.0110 (0.0018)	−0.0096 (0.0062)	0.0091 (0.0141)	0.0319 (0.0277)	−0.0129 (0.0017)	−0.0015 (0.0053)	−0.0006 (0.0115)	0.0651 (0.0285)
	U	−0.0140 (0.0020)	−0.0095 (0.0066)	0.0033 (0.0138)	0.0270 (0.0270)	−0.0153 (0.0018)	0.0109 (0.0062)	−0.0150 (0.0084)	0.0753 (0.0276)
	M	−0.0093 (0.0020)	−0.0118 (0.0072)	0.0121 (0.0153)	0.0249 (0.0294)	−0.0144 (0.0018)	0.0177 (0.0067)	−0.0372 (0.0074)	0.1040 (0.0309)
Non-information Bayes									
(50,30)	L	−0.0261 (0.0050)	−0.0303 (0.0177)	0.0545 (0.0476)	0.0705 (0.0919)	−0.0262 (0.0039)	0.0040 (0.0148)	−0.0138 (0.0123)	0.1572 (0.0834)
	R	−0.0083 (0.0039)	−0.0181 (0.0112)	0.0540 (0.0383)	0.0555 (0.0579)	−0.0077 (0.0043)	−0.0117 (0.0115)	0.0600 (0.0433)	0.0669 (0.0594)
	U	−0.0191 (0.0038)	−0.0211 (0.0124)	0.0326 (0.0335)	0.0515 (0.0557)	−0.0175 (0.0038)	−0.0078 (0.0114)	0.0286 (0.0278)	0.0793 (0.0604)
	M	−0.0194 (0.0042)	−0.0242 (0.0142)	0.0123 (0.0315)	0.0318 (0.0596)	−0.0219 (0.0034)	0.0079 (0.0124)	−0.0230 (0.0142)	0.1124 (0.0563)
(80,30)	L	−0.0253 (0.0054)	−0.0302 (0.0170)	0.0513 (0.0487)	0.0690 (0.0955)	−0.0263 (0.0040)	0.0094 (0.0150)	−0.0189 (0.0122)	0.1461 (0.0799)
	R	0.0206 (0.0081)	−0.0093 (0.0178)	0.0705 (0.0518)	0.0047 (0.0484)	0.0227 (0.0100)	−0.0119 (0.0176)	0.0789 (0.0547)	0.0087 (0.0488)
	U	−0.0073 (0.0040)	−0.0170 (0.0114)	0.0383 (0.0297)	0.0017 (0.0389)	−0.0052 (0.0041)	−0.0119 (0.0117)	0.0347 (0.0286)	0.0210 (0.0375)
	M	−0.0184 (0.0047)	−0.0275 (0.0171)	−0.0017 (0.0228)	−0.0246 (0.0394)	−0.0247 (0.0034)	0.0112 (0.0117)	−0.0270 (0.0120)	0.0670 (0.0390)
(80,60)	L	−0.0158 (0.0023)	−0.0160 (0.0081)	0.0156 (0.0193)	0.0386 (0.0391)	−0.0160 (0.0020)	0.0196 (0.0082)	−0.0386 (0.0064)	0.1271 (0.0415)
	R	−0.0123 (0.0017)	−0.0056 (0.0061)	0.0077 (0.0141)	0.0370 (0.0277)	−0.0132 (0.0017)	0.0038 (0.0055)	−0.0017 (0.0107)	0.0567 (0.0272)
	U	−0.0130 (0.0020)	−0.0122 (0.0065)	0.0087 (0.0154)	0.0344 (0.0304)	−0.0153 (0.0018)	0.0086 (0.0060)	−0.0111 (0.0088)	0.0823 (0.0299)
	M	−0.0127 (0.0022)	−0.0110 (0.0071)	0.0038 (0.0156)	0.0211 (0.0308)	−0.0148 (0.0017)	0.0167 (0.0072)	−0.0350 (0.0071)	0.1076 (0.0345)

**Table A3:** ABs and MSEs (within bracket) for MLE and Bayes point estimation of parameters under ($T_{1}=100,T_{2}=101$).

(n,m)	CS	$T_{1}=100,T_{2}=101$			
		${\hat{\alpha }}_{1}$	${\hat{\alpha }}_{2}$	${\hat{\beta }}_{1}$	${\hat{\beta }}_{2}$
MLE					
(50,30)	L	−0.0090 (0.0039)	0.0292 (0.0200)	−0.0285 (0.0159)	0.0425 (0.1185)
	R	0.0098 (0.0048)	0.0180 (0.0128)	0.0640 (0.0421)	0.0711 (0.0635)
	U	−0.0018 (0.0036)	0.0260 (0.0131)	0.0122 (0.0208)	0.0860 (0.0557)
	M	−0.0053 (0.0034)	0.0380 (0.0161)	−0.0242 (0.0167)	0.0841 (0.0782)
(80,30)	L	−0.0110 (0.0039)	−0.0136 (0.0369)	−0.0318 (0.0173)	−0.1761 (0.2561)
	R	0.0467 (0.0142)	0.0171 (0.0195)	0.1208 (0.0730)	0.0345 (0.0580)
	U	0.0130 (0.0054)	0.0235 (0.0143)	0.0483 (0.0304)	0.0451 (0.0433)
	M	−0.0070 (0.0035)	0.0288 (0.0233)	−0.0257 (0.0198)	−0.0354 (0.1568)
(80,60)	L	−0.0075 (0.0018)	0.0332 (0.0093)	−0.0400 (0.0068)	0.1186 (0.0399)
	R	−0.0039 (0.0017)	0.0159 (0.0058)	−0.0027 (0.0106)	0.0539 (0.0267)
	U	−0.0071 (0.0017)	0.0253 (0.0069)	−0.0216 (0.0078)	0.0783 (0.0290)
	M	−0.0066 (0.0017)	0.0338 (0.0079)	−0.0360 (0.0067)	0.1067 (0.0324)
Information Bayes					
(50,30)	L	−0.0253 (0.0038)	0.0069 (0.0139)	−0.0202 (0.0118)	0.1522 (0.0761)
	R	−0.0062 (0.0038)	−0.0111 (0.0103)	0.0644 (0.0378)	0.0551 (0.0520)
	U	−0.0221 (0.0033)	0.0040 (0.0111)	−0.0260 (0.0122)	0.1051 (0.0507)
	M	−0.0193 (0.0032)	−0.0011 (0.0110)	0.0065 (0.0192)	0.0818 (0.0512)
(80,30)	L	−0.0273 (0.0040)	0.0088 (0.0140)	−0.0118 (0.0114)	0.1391 (0.0712)
	R	0.0140 (0.0066)	−0.0166 (0.0135)	0.0648 (0.0410)	0.0052 (0.0435)
	U	−0.0049 (0.0043)	−0.0091 (0.0111)	0.0295 (0.0242)	0.0245 (0.0337)
	M	−0.0236 (0.0032)	0.0139 (0.0126)	−0.0218 (0.0113)	0.0628 (0.0330)
(80,60)	L	−0.0173 (0.0020)	0.0164 (0.0074)	−0.0358 (0.0064)	0.1258 (0.0397)
	R	−0.0128 (0.0017)	0.0010 (0.0053)	−0.0039 (0.0104)	0.0545 (0.0270)
	U	−0.0140 (0.0018)	0.0101 (0.0058)	−0.0225 (0.0082)	0.0788 (0.0290)
	M	−0.0147 (0.0017)	0.0153 (0.0067)	−0.0399 (0.0065)	0.1067 (0.0322)
Non-information Bayes					
(50,30)	L	−0.0235 (0.0043)	0.0085 (0.0156)	−0.0177 (0.0120)	0.1485 (0.0759)
	R	−0.0075 (0.0039)	−0.0085 (0.0110)	0.0622 (0.0399)	0.0723 (0.0608)
	U	−0.0182 (0.0035)	−0.0039 (0.0114)	0.0099 (0.0208)	0.1015 (0.0620)
	M	−0.0212 (0.0035)	0.0051 (0.0123)	−0.0214 (0.0135)	0.1154 (0.0579)
(80,30)	L	−0.0269 (0.0039)	0.0040 (0.0149)	−0.0175 (0.0118)	0.1587 (0.0878)
	R	0.0232 (0.0095)	−0.0105 (0.0187)	0.0745 (0.0487)	0.0058 (0.0491)
	U	−0.0112 (0.0041)	−0.0102 (0.0124)	0.0328 (0.0274)	0.0357 (0.0395)
	M	−0.0274 (0.0035)	0.0158 (0.0132)	−0.0203 (0.0123)	0.0639 (0.0316)
(80,60)	L	−0.0162 (0.0020)	0.0146 (0.0076)	−0.0397 (0.0066)	0.1365 (0.0443)
	R	−0.0125 (0.0018)	0.0044 (0.0053)	0.0001 (0.0111)	0.0564 (0.0278)
	U	−0.0146 (0.0018)	0.0091 (0.0060)	−0.0177 (0.0081)	0.0813 (0.0298)
	M	−0.0137 (0.0017)	0.0158 (0.0067)	−0.0405 (0.0068)	0.1097 (0.0335)

**Table A4:** ILs and CPs (within bracket) with ($T_{1}=0.8,T_{2}=1.2$).

** (n,m) **	**CS**	**ACIS**				**HPD**			
		** ${\hat{\alpha }}_{1}$ **	** ${\hat{\alpha }}_{2}$ **	** ${\hat{\beta }}_{1}$ **	** ${\hat{\beta }}_{2}$ **	** ${\hat{\alpha }}_{1}$ **	** ${\hat{\alpha }}_{2}$ **	** ${\hat{\beta }}_{1}$ **	** ${\hat{\beta }}_{2}$ **
(50,30)	L	0.3600 (0.9360)	0.5947 (0.9380)	1.1700 (0.9220)	1.0640 (0.9260)	0.2375 (0.8525)	0.4569 (0.8800)	0.8103 (0.9615)	0.9038 (0.9455)
	R	0.2520 (0.9440)	0.4490 (0.9540)	0.8480 (0.9350)	0.9720 (0.9160)	0.2096 (0.8985)	0.3860 (0.9180)	0.6890 (0.9545)	0.7627 (0.9205)
	U	0.2800 (0.9370)	0.5070 (0.9400)	0.9030 (0.9270)	0.9160 (0.9100)	0.2164 (0.8805)	0.4058 (0.9025)	0.7033 (0.9535)	0.7813 (0.9220)
	M	0.4220 (0.9310)	0.6360 (0.9480)	1.3700 (0.9180)	1.1170 (0.9240)	0.2399 (0.8890)	0.4413 (0.8930)	0.7347 (0.9595)	0.8060 (0.9240)
(80,30)	L	0.3116 (0.9407)	0.5383 (0.9433)	1.0004 (0.9296)	0.8322 (0.9472)	0.2390 (0.8555)	0.4578 (0.8705)	0.7735 (0.9505)	0.8326 (0.9205)
	R	0.2310 (0.8215)	0.3650 (0.9287)	0.6990 (0.8113)	0.7658 (0.9130)	0.2764 (0.9285)	0.4202 (0.9015)	0.7306 (0.9575)	0.7081 (0.9200)
	U	0.2533 (0.9395)	0.5086 (0.9583)	0.8336 (0.9375)	0.8300 (0.9258)	0.2192 (0.9020)	0.3983 (0.9005)	0.6271 (0.9485)	0.6623 (0.9120)
	M	0.3201 (0.9156)	0.4355 (0.9416)	0.8689 (0.9107)	1.0701 (0.9345)	0.2517 (0.8920)	0.4542 (0.8800)	0.6572 (0.9360)	0.6999 (0.8980)
(80,60)	L	0.2115 (0.9470)	0.3917 (0.9442)	0.6677 (0.9311)	0.7144 (0.9034)	0.1812 (0.8975)	0.3419 (0.9120)	0.5782 (0.9440)	0.6510 (0.9305)
	R	0.1788 (0.9431)	0.3315 (0.9474)	0.5743 (0.9380)	0.6239 (0.9135)	0.1587 (0.9065)	0.3001 (0.9275)	0.5138 (0.9475)	0.5768 (0.9145)
	U	0.1932 (0.9440)	0.3570 (0.9468)	0.6055 (0.9310)	0.6496 (0.9066)	0.1685 (0.9100)	0.3186 (0.9145)	0.5375 (0.9415)	0.5936 (0.9130)
	M	0.2071 (0.9334)	0.3722 (0.9454)	0.6538 (0.9325)	0.6860 (0.9105)	0.1766 (0.9180)	0.3283 (0.9330)	0.5501 (0.9460)	0.6035 (0.9245)
** (n,m) **	**CS**	**Boot-p**				**Boot-t**			
		** ${\hat{\alpha }}_{1}$ **	** ${\hat{\alpha }}_{2}$ **	** ${\hat{\beta }}_{1}$ **	** ${\hat{\beta }}_{2}$ **	** ${\hat{\alpha }}_{1}$ **	** ${\hat{\alpha }}_{2}$ **	** ${\hat{\beta }}_{1}$ **	** ${\hat{\beta }}_{2}$ **
(50,30)	L	0.2364 (1.0000)	0.2214 (1.0000)	0.2991 (1.0000)	0.2744 (0.9980)	0.2685 (1.0000)	0.5063 (1.0000)	0.7762 (1.0000)	0.8692 (1.0000)
	R	0.5039 (1.0000)	0.3743 (1.0000)	0.5565 (0.9840)	0.4595 (1.0000)	0.1864 (1.0000)	0.3361 (1.0000)	0.5282 (1.0000)	0.7670 (1.0000)
	U	1.2512 (0.9920)	0.8606 (1.0000)	0.8892 (1.0000)	0.8282 (1.0000)	0.2726 (1.0000)	0.4883 (1.0000)	0.5691 (1.0000)	0.8701 (1.0000)
	M	1.1286 (0.9580)	0.7488 (0.9940)	0.9141 (0.9940)	0.7010 (0.9980)	0.2745 (0.9960)	0.4995 (1.0000)	0.6955 (0.9960)	0.8288 (1.0000)
(80,30)	L	0.2863 (1.0000)	0.4433 (0.9940)	0.3655 (0.9900)	0.2691 (0.9980)	0.2660 (1.0000)	0.4637 (1.0000)	0.8074 (0.9960)	0.6067 (1.0000)
	R	0.7536 (0.9980)	0.5553 (1.0000)	0.6033 (1.0000)	0.4823 (0.9740)	0.0771 (0.9840)	0.5783 (0.9880)	0.3730 (0.7440)	0.8848 (1.0000)
	U	1.5328 (1.0000)	0.8408 (0.9900)	0.8363 (1.0000)	0.7018 (0.9900)	0.2447 (1.0000)	0.3559 (0.9860)	0.7898 (0.9920)	0.7090 (0.9940)
	M	0.9488 (0.9960)	0.9625 (0.9500)	0.6847 (1.0000)	0.7469 (0.9840)	0.2595 (1.0000)	0.4399 (0.9840)	0.6665 (0.9780)	0.6828 (1.0000)
(80,60)	L	0.1776 (1.0000)	0.1510 (1.0000)	0.1739 (1.0000)	0.1619 (1.0000)	0.1836 (1.0000)	0.3111 (1.0000)	0.5507 (1.0000)	0.7260 (1.0000)
	R	0.3283 (1.0000)	0.2871 (0.9980)	0.3508 (0.9420)	0.3139 (0.9960)	0.1520 (1.0000)	0.2640 (1.0000)	0.4429 (1.0000)	0.5981 (0.9980)
	U	0.6873 (1.0000)	0.5616 (1.0000)	0.6050 (0.9960)	0.6065 (0.9980)	0.1592 (1.0000)	0.3025 (1.0000)	0.4845 (1.0000)	0.5834 (1.0000)
	M	0.5845 (0.9940)	0.4807 (1.0000)	0.6010 (0.9980)	0.5171 (1.0000)	0.1759 (1.0000)	0.3154 (0.9920)	0.5168 (0.9920)	0.6382 (1.0000)

**Table A5:** ILs and CPs (within bracket) with ($T_{1}=0.8,T_{2}=1.5$).

** (n,m) **	**CS**	**ACIS**				**HPD**			
		** ${\hat{\alpha }}_{1}$ **	** ${\hat{\alpha }}_{2}$ **	** ${\hat{\beta }}_{1}$ **	** ${\hat{\beta }}_{2}$ **	** ${\hat{\alpha }}_{1}$ **	** ${\hat{\alpha }}_{2}$ **	** ${\hat{\beta }}_{1}$ **	** ${\hat{\beta }}_{2}$ **
(50,30)	L	0.2478 (0.9429)	0.4673 (0.9389)	0.7582 (0.9342)	0.8335 (0.9015)	0.2236 (0.8170)	0.4470 (0.8795)	0.7497 (0.9520)	0.8757 (0.9080)
	R	0.3048 (0.9322)	0.6060 (0.9437)	0.8979 (0.9109)	0.9813 (0.9041)	0.2078 (0.8860)	0.3866 (0.9220)	0.6953 (0.9475)	0.7715 (0.9255)
	U	0.2897 (0.9403)	0.5436 (0.9381)	0.9412 (0.9404)	0.9562 (0.9562)	0.2062 (0.8750)	0.4016 (0.9080)	0.6443 (0.9410)	0.7558 (0.9145)
	M	0.2440 (0.9453)	0.4352 (0.9518)	0.7916 (0.9393)	0.8311 (0.9194)	0.2223 (0.8580)	0.4287 (0.8895)	0.6761 (0.9460)	0.7998 (0.9110)
(80,30)	L	0.2624 (0.9449)	0.4959 (0.9403)	0.7881 (0.9415)	0.7576 (0.9500)	0.2229 (0.8475)	0.4367 (0.8910)	0.6782 (0.9360)	0.8120 (0.9150)
	R	0.2751 (0.8222)	0.4175 (0.9329)	0.6150 (0.8118)	0.5855 (0.9156)	0.2783 (0.9430)	0.4248 (0.9105)	0.7316 (0.9525)	0.7132 (0.9215)
	U	0.2446 (0.9363)	0.4803 (0.9597)	0.4828 (0.9352)	0.7974 (0.9267)	0.2099 (0.8955)	0.3910 (0.9110)	0.5966 (0.9465)	0.6546 (0.9190)
	M	0.6022 (0.9330)	0.8281 (0.9487)	1.2201 (0.9210)	1.0026 (0.9302)	0.2274 (0.8755)	0.4431 (0.9040)	0.5901 (0.9425)	0.6700 (0.9095)
(80,60)	L	0.1921 (0.9455)	0.3659 (0.9421)	0.5757 (0.9414)	0.6603 (0.9084)	0.1695 (0.8910)	0.3275 (0.9220)	0.5096 (0.9500)	0.6026 (0.9205)
	R	0.1645 (0.9495)	0.3122 (0.9478)	0.4980 (0.9497)	0.5760 (0.8987)	0.1504 (0.9155)	0.2878 (0.9260)	0.4501 (0.9410)	0.5454 (0.9170)
	U	0.1760 (0.9465)	0.3360 (0.9416)	0.5232 (0.9458)	0.6009 (0.9011)	0.1591 (0.9030)	0.3039 (0.9255)	0.4685 (0.9475)	0.5707 (0.9290)
	M	0.1863 (0.9408)	0.3491 (0.9527)	0.5520 (0.9336)	0.6314 (0.8967)	0.1644 (0.8965)	0.3141 (0.9225)	0.4877 (0.9545)	0.5763 (0.9200)
** (n,m) **	**CS**	**Boot-p**				**Boot-t**			
		** ${\hat{\alpha }}_{1}$ **	** ${\hat{\alpha }}_{2}$ **	** ${\hat{\beta }}_{1}$ **	** ${\hat{\beta }}_{2}$ **	** ${\hat{\alpha }}_{1}$ **	** ${\hat{\alpha }}_{2}$ **	** ${\hat{\beta }}_{1}$ **	** ${\hat{\beta }}_{2}$ **
(50,30)	L	0.2622 (1.0000)	0.2146 (1.0000)	0.2616 (1.0000)	0.2182 (1.0000)	0.2706 (1.0000)	0.4847 (1.0000)	0.6099 (1.0000)	0.6987 (1.0000)
	R	0.5058 (1.0000)	0.3376 (1.0000)	0.4567 (1.0000)	0.4216 (0.9980)	0.2120 (1.0000)	0.3891 (1.0000)	0.6510 (1.0000)	0.6497 (1.0000)
	U	0.8708 (1.0000)	0.8357 (1.0000)	0.8746 (1.0000)	0.7873 (0.9980)	0.1966 (1.0000)	0.3804 (1.0000)	0.6785 (1.0000)	0.6576 (0.9980)
	M	0.7224 (1.0000)	0.6200 (1.0000)	0.7150 (1.0000)	0.6568 (1.0000)	0.5356 (1.0000)	0.4502 (0.9940)	1.5390 (1.0000)	0.7375 (1.0000)
(80,30)	L	0.1966 (0.8580)	0.4311 (0.9980)	0.3129 (1.0000)	0.2642 (1.0000)	0.2493 (1.0000)	0.4701 (1.0000)	0.5538 (1.0000)	0.6510 (1.0000)
	R	0.6653 (0.9980)	0.5521 (1.0000)	0.5740 (0.9980)	0.4269 (1.0000)	0.2543 (1.0000)	0.3171 (1.0000)	0.9909 (1.0000)	0.6641 (1.0000)
	U	1.4076 (1.0000)	0.7486 (1.0000)	0.8805 (0.9980)	0.7115 (0.9960)	0.2032 (1.0000)	0.3718 (1.0000)	0.5168 (1.0000)	0.5679 (1.0000)
	M	1.0535 (1.0000)	0.8107 (1.0000)	0.6539 (0.9880)	0.6004 (1.0000)	0.3305 (1.0000)	0.5386 (0.9900)	1.0490 (1.0000)	0.7450 (1.0000)
(80,60)	L	0.1479 (1.0000)	0.1353 (1.0000)	0.1641 (1.0000)	0.1398 (0.9980)	0.1564 (1.0000)	0.2913 (1.0000)	0.5283 (0.9860)	0.6693 (0.9900)
	R	0.2924 (1.0000)	0.2596 (1.0000)	0.3005 (1.0000)	0.2925 (0.9980)	0.1422 (1.0000)	0.2590 (1.0000)	0.4319 (1.0000)	0.4916 (1.0000)
	U	0.6946 (0.9900)	0.5627 (1.0000)	0.5859 (1.0000)	0.5616 (1.0000)	0.1493 (1.0000)	0.2853 (1.0000)	0.3707 (1.0000)	0.6007 (1.0000)
	M	0.5197 (0.9940)	0.4170 (1.0000)	0.4463 (1.0000)	0.4577 (1.0000)	0.1509 (1.0000)	0.2768 (1.0000)	0.4569 (1.0000)	0.5909 (1.0000)

**Table A6:** ILs and CPs (within bracket) with ($T_{1}=1,T_{2}=1.5$).

** (n,m) **	**CS**	**ACIS**				**HPD**			
		** ${\hat{\alpha }}_{1}$ **	** ${\hat{\alpha }}_{2}$ **	** ${\hat{\beta }}_{1}$ **	** ${\hat{\beta }}_{2}$ **	** ${\hat{\alpha }}_{1}$ **	** ${\hat{\alpha }}_{2}$ **	** ${\hat{\beta }}_{1}$ **	** ${\hat{\beta }}_{2}$ **
(50,30)	L	0.2877 (0.9490)	0.5432 (0.9348)	0.9209 (0.9411)	0.9581 (0.9217)	0.2222 (0.8265)	0.4410 (0.8945)	0.7261 (0.9560)	0.8447 (0.9265)
	R	0.2394 (0.9447)	0.4342 (0.9525)	0.7793 (0.9439)	0.8273 (0.9123)	0.2059 (0.8970)	0.3844 (0.9265)	0.6630 (0.9505)	0.7494 (0.9385)
	U	0.2537 (0.9380)	0.4755 (0.9368)	0.7696 (0.9301)	0.8426 (0.8985)	0.2054 (0.8740)	0.3976 (0.9130)	0.6302 (0.9500)	0.7413 (0.9215)
	M	0.3060 (0.9353)	0.5518 (0.9391)	0.8978 (0.9123)	0.9574 (0.9014)	0.2198 (0.8795)	0.4238 (0.8995)	0.6627 (0.9550)	0.7767 (0.9175)
(80,30)	L	0.2602 (0.9453)	0.4935 (0.9430)	0.7685 (0.9337)	0.7631 (0.9482)	0.2206 (0.8275)	0.4390 (0.8725)	0.6833 (0.9525)	0.7996 (0.9295)
	R	0.2132 (0.8263)	0.3921 (0.9317)	0.5293 (0.8188)	0.6157 (0.9147)	0.2744 (0.9485)	0.4208 (0.8945)	0.7303 (0.9580)	0.7210 (0.9285)
	U	0.2806 (0.9421)	0.6167 (0.9606)	0.7678 (0.9269)	0.8658 (0.9200)	0.2133 (0.8970)	0.3939 (0.9120)	0.5947 (0.9475)	0.6527 (0.9105)
	M	0.7370 (0.9351)	0.3705 (0.9420)	1.5899 (0.9169)	8.1600 (0.9339)	0.2287 (0.8830)	0.4397 (0.8875)	0.5977 (0.9480)	0.6718 (0.9035)
(80,60)	L	0.1926 (0.9490)	0.3659 (0.9417)	0.5732 (0.9407)	0.6604 (0.8996)	0.1702 (0.8950)	0.3276 (0.9130)	0.5079 (0.9395)	0.6119 (0.9090)
	R	0.1648 (0.9513)	0.3122 (0.9424)	0.4993 (0.9492)	0.5803 (0.9080)	0.1505 (0.9010)	0.2875 (0.9235)	0.4521 (0.9415)	0.5457 (0.9120)
	U	0.1754 (0.9481)	0.3357 (0.9393)	0.5241 (0.9396)	0.5986 (0.9020)	0.1579 (0.8995)	0.3054 (0.9300)	0.4679 (0.9460)	0.5613 (0.9190)
	M	0.1871 (0.9423)	0.3517 (0.9406)	0.5560 (0.9347)	0.6308 (0.9091)	0.1654 (0.9195)	0.3141 (0.9255)	0.4900 (0.9520)	0.5799 (0.9205)
** (n,m) **	**CS**	**Boot-p**				**Boot-t**			
		** ${\hat{\alpha }}_{1}$ **	** ${\hat{\alpha }}_{2}$ **	** ${\hat{\beta }}_{1}$ **	** ${\hat{\beta }}_{2}$ **	** ${\hat{\alpha }}_{1}$ **	** ${\hat{\alpha }}_{2}$ **	** ${\hat{\beta }}_{1}$ **	** ${\hat{\beta }}_{2}$ **
(50,30)	L	0.2548 (1.0000)	0.2534 (0.9900)	0.2759 (0.9980)	0.2453 (1.0000)	0.2512 (0.9980)	0.4062 (0.9980)	0.6512 (0.9960)	0.7920 (1.0000)
	R	0.5200 (1.0000)	0.3504 (0.9980)	0.5183 (0.9860)	0.4288 (1.0000)	0.2323 (1.0000)	0.3864 (0.9880)	0.5773 (1.0000)	0.7269 (1.0000)
	U	1.2183 (0.9940)	0.9746 (1.0000)	0.8334 (1.0000)	0.8242 (1.0000)	0.2959 (1.0000)	0.4673 (1.0000)	0.6855 (0.9980)	0.8261 (0.9900)
	M	0.7746 (0.9840)	0.6705 (0.9980)	0.7298 (0.9980)	0.6162 (0.9980)	0.2499 (0.9560)	0.4670 (1.0000)	0.6221 (1.0000)	0.7308 (0.9980)
(80,30)	L	0.2418 (1.0000)	0.4559 (0.9840)	0.3595 (0.8360)	0.2722 (0.9900)	0.2291 (1.0000)	0.4352 (1.0000)	0.7256 (1.0000)	0.5586 (1.0000)
	R	0.6627 (0.9320)	0.5482 (1.0000)	0.5978 (1.0000)	0.4237 (1.0000)	0.2846 (1.0000)	0.3244 (1.0000)	1.1983 (0.9940)	0.7802 (1.0000)
	U	0.9929 (0.9980)	0.8102 (1.0000)	1.1573 (0.9900)	0.7368 (1.0000)	0.2011 (1.0000)	0.3760 (1.0000)	0.5573 (1.0000)	0.5844 (1.0000)
	M	0.8234 (1.0000)	0.8218 (0.9960)	0.6237 (0.9160)	0.6056 (1.0000)	0.2697 (1.0000)	0.4716 (1.0000)	0.6427 (1.0000)	0.6160 (1.0000)
(80,60)	L	0.1735 (1.0000)	0.1295 (0.9920)	0.1651 (0.9980)	0.1535 (0.9980)	0.1642 (1.0000)	0.3064 (1.0000)	0.4671 (1.0000)	0.5534 (0.9980)
	R	0.2995 (1.0000)	0.2545 (1.0000)	0.2815 (1.0000)	0.2837 (1.0000)	0.1386 (1.0000)	0.2594 (1.0000)	0.4164 (1.0000)	0.4951 (1.0000)
	U	0.6949 (1.0000)	0.5792 (1.0000)	0.6434 (1.0000)	0.5647 (1.0000)	0.1473 (1.0000)	0.2795 (1.0000)	0.4174 (1.0000)	0.5373 (1.0000)
	M	0.4547 (1.0000)	0.4449 (1.0000)	0.4491 (1.0000)	0.4206 (1.0000)	0.1545 (1.0000)	0.2815 (1.0000)	0.5306 (0.9940)	0.6319 (0.9940)

**Table A7:** ILs and CPs (within bracket) with ($T_{1}=0.8,T_{2}=100$).

** (n,m) **	**CS**	**ACIS**				**HPD**			
		** ${\hat{\alpha }}_{1}$ **	** ${\hat{\alpha }}_{2}$ **	** ${\hat{\beta }}_{1}$ **	** ${\hat{\beta }}_{2}$ **	** ${\hat{\alpha }}_{1}$ **	** ${\hat{\alpha }}_{2}$ **	** ${\hat{\beta }}_{1}$ **	** ${\hat{\beta }}_{2}$ **
(50,30)	L	0.2032 (1.0000)	0.4495 (1.0000)	0.3741 (1.0000)	1.3157 (0.9988)	0.2104 (0.8825)	0.4261 (0.9290)	0.3805 (0.9175)	0.7112 (0.8840)
	R	0.2390 (0.9420)	0.4316 (0.9552)	0.7787 (0.9443)	0.8254 (0.9106)	0.2064 (0.9040)	0.3828 (0.9205)	0.6497 (0.9535)	0.7552 (0.9275)
	U	0.2346 (0.9570)	0.4361 (0.9405)	0.6450 (0.9500)	0.7526 (0.9030)	0.1952 (0.8680)	0.3874 (0.9235)	0.5556 (0.9405)	0.7071 (0.9390)
	M	0.2557 (0.9682)	0.4505 (0.9276)	0.4501 (0.9662)	0.6844 (0.8826)	0.1973 (0.8745)	0.4016 (0.9410)	0.3920 (0.9140)	0.6601 (0.9075)
(80,30)	L	0.1935 (0.9312)	0.4271 (0.9123)	0.3837 (0.9162)	0.6260 (0.9639)	0.2021 (0.8400)	0.4261 (0.9245)	0.3726 (0.9330)	0.6787 (0.8665)
	R	0.2247 (0.8202)	0.3612 (0.9323)	0.4938 (0.8123)	0.5948 (0.9128)	0.2783 (0.9325)	0.4267 (0.9155)	0.7362 (0.9600)	0.7158 (0.9160)
	U	0.1817 (0.9417)	0.4732 (0.9604)	0.4666 (0.9365)	0.7466 (0.9231)	0.2133 (0.8925)	0.3946 (0.9360)	0.5919 (0.9505)	0.6522 (0.9145)
	M	0.2017 (0.9590)	0.4361 (0.9304)	0.3646 (0.9569)	0.5515 (0.9185)	0.1912 (0.8625)	0.4113 (0.9450)	0.3784 (0.9095)	0.5783 (0.9205)
(80,60)	L	0.3047 (0.9673)	0.3628 (0.9240)	0.4165 (0.9472)	0.6399 (0.9316)	0.1582 (0.9035)	0.3164 (0.9325)	0.2681 (0.8800)	0.5027 (0.8205)
	R	0.1592 (0.9617)	0.2985 (0.9440)	0.4407 (0.9609)	0.5379 (0.9063)	0.1456 (0.9010)	0.2804 (0.9405)	0.3904 (0.9295)	0.5196 (0.9145)
	U	0.1691 (0.9722)	0.3090 (0.9346)	0.4131 (0.9631)	0.5321 (0.8919)	0.1498 (0.8945)	0.2943 (0.9410)	0.3407 (0.9295)	0.4982 (0.9000)
	M	0.1696 (0.9674)	0.3184 (0.9210)	0.3331 (0.9515)	0.5225 (0.8625)	0.1502 (0.9050)	0.2975 (0.9430)	0.2775 (0.8775)	0.4827 (0.8730)
** (n,m) **	**CS**	**Boot-p**				**Boot-t**			
		** ${\hat{\alpha }}_{1}$ **	** ${\hat{\alpha }}_{2}$ **	** ${\hat{\beta }}_{1}$ **	** ${\hat{\beta }}_{2}$ **	** ${\hat{\alpha }}_{1}$ **	** ${\hat{\alpha }}_{2}$ **	** ${\hat{\beta }}_{1}$ **	** ${\hat{\beta }}_{2}$ **
(50,30)	L	0.2295 (1.0000)	0.2221 (1.0000)	0.1933 (1.0000)	0.2557 (0.9940)	0.2236 (1.0000)	0.2957 (0.9900)	0.3400 (1.0000)	0.5004 (0.9380)
	R	0.4873 (1.0000)	0.3745 (1.0000)	0.3522 (0.9920)	0.3962 (1.0000)	0.2044 (1.0000)	0.3773 (1.0000)	0.6649 (1.0000)	0.6593 (1.0000)
	U	0.9884 (1.0000)	0.7617 (1.0000)	1.1604 (0.9960)	0.7725 (0.9960)	0.1947 (1.0000)	0.3680 (1.0000)	0.4760 (1.0000)	0.5956 (1.0000)
	M	0.3605 (1.0000)	0.6784 (1.0000)	0.3718 (1.0000)	0.5026 (0.9980)	0.1734 (1.0000)	0.3639 (1.0000)	0.3457 (1.0000)	0.6118 (0.9960)
(80,30)	L	0.2193 (0.9980)	0.5551 (1.0000)	0.3648 (1.0000)	1.3438 (1.0000)	0.1707 (0.9380)	0.3448 (0.9340)	0.3303 (0.9380)	0.5932 (0.9360)
	R	0.3460 (1.0000)	0.4687 (0.9980)	0.8840 (1.0000)	0.7666 (1.0000)	0.1181 (0.9260)	0.4521 (0.9380)	0.3265 (0.9380)	0.7909 (0.9380)
	U	0.2194 (0.9940)	0.4581 (1.0000)	0.3456 (1.0000)	1.3719 (1.0000)	0.1765 (0.9360)	0.3232 (0.9360)	0.4787 (0.9360)	0.5463 (0.9360)
	M	0.2567 (1.0000)	0.4114 (1.0000)	0.6190 (0.9960)	0.7021 (1.0000)	0.1893 (0.9080)	0.3621 (0.9360)	0.3375 (0.9360)	0.4818 (0.9360)
(80,60)	L	0.1459 (1.0000)	0.1528 (0.9820)	0.1428 (0.9980)	0.1290 (0.9980)	0.1418 (1.0000)	0.2617 (1.0000)	0.2292 (0.9980)	0.5130 (0.9880)
	R	0.2806 (1.0000)	0.2375 (1.0000)	0.2570 (1.0000)	0.2528 (1.0000)	0.1370 (1.0000)	0.2511 (1.0000)	0.3570 (1.0000)	0.4857 (1.0000)
	U	0.5801 (0.9700)	0.5309 (1.0000)	0.5156 (0.9940)	0.5250 (0.9760)	0.1408 (0.9980)	0.2512 (1.0000)	0.2794 (1.0000)	0.4655 (1.0000)
	M	0.2289 (0.9980)	0.3132 (1.0000)	0.2288 (0.9980)	0.3053 (1.0000)	0.1405 (1.0000)	0.2635 (1.0000)	0.2297 (0.9900)	0.5468 (1.0000)

**Table A8:** ILs and CPs (within bracket) with ($T_{1}=100,T_{2}=101$).

** (n,m) **	**CS**	**ACIS**				**HPD**			
		** ${\hat{\alpha }}_{1}$ **	** ${\hat{\alpha }}_{2}$ **	** ${\hat{\beta }}_{1}$ **	** ${\hat{\beta }}_{2}$ **	** ${\hat{\alpha }}_{1}$ **	** ${\hat{\alpha }}_{2}$ **	** ${\hat{\beta }}_{1}$ **	** ${\hat{\beta }}_{2}$ **
(50,30)	L	0.2381 (0.9820)	0.4396 (1.0000)	0.3428 (1.0000)	1.4519 (1.0000)	0.2095 (0.8685)	0.4280 (0.9195)	0.3835 (0.9080)	0.7111 (0.8775)
	R	0.2414 (0.9394)	0.4367 (0.9525)	0.7812 (0.9442)	0.8351 (0.9140)	0.2076 (0.9010)	0.3829 (0.9270)	0.6731 (0.9540)	0.7435 (0.9235)
	U	0.2371 (0.9596)	0.4431 (0.9492)	0.5812 (0.9950)	0.7670 (0.9113)	0.1969 (0.8810)	0.3917 (0.9270)	0.4961 (0.9435)	0.6944 (0.9260)
	M	0.2320 (0.9675)	0.4482 (0.9330)	0.4442 (0.9618)	0.6833 (0.8872)	0.1967 (0.8730)	0.3996 (0.9415)	0.3898 (0.9095)	0.6556 (0.8970)
(80,30)	L	0.2803 (0.9312)	0.4204 (0.9048)	0.4854 (0.9190)	0.6176 (0.9616)	0.2039 (0.8475)	0.4258 (0.9205)	0.3727 (0.9225)	0.6850 (0.8920)
	R	0.1842 (0.8245)	0.3393 (0.9333)	0.4710 (0.8144)	0.6346 (0.9147)	0.2740 (0.9355)	0.4260 (0.9025)	0.7309 (0.9585)	0.7202 (0.9145)
	U	0.2037 (0.9344)	0.4671 (0.9595)	0.4987 (0.9383)	0.7383 (0.9236)	0.2123 (0.8925)	0.3974 (0.9385)	0.5557 (0.9440)	0.6458 (0.9375)
	M	0.2021 (0.9580)	0.4369 (0.9247)	0.3655 (0.9584)	0.5509 (0.9133)	0.1895 (0.8685)	0.4114 (0.9420)	0.3754 (0.9265)	0.5758 (0.9145)
(80,60)	L	0.2685 (0.9678)	0.4106 (0.9230)	0.4179 (0.9475)	0.6967 (0.9300)	0.1585 (0.8965)	0.3155 (0.9380)	0.2679 (0.8845)	0.5040 (0.8435)
	R	0.1591 (0.9579)	0.3011 (0.9496)	0.4418 (0.9611)	0.5513 (0.9106)	0.1457 (0.9035)	0.2817 (0.9380)	0.3892 (0.9285)	0.5123 (0.9135)
	U	0.1705 (0.9705)	0.3172 (0.9380)	0.3996 (0.9640)	0.5504 (0.9080)	0.1505 (0.9005)	0.2946 (0.9460)	0.3230 (0.9095)	0.4972 (0.8955)
	M	0.1694 (0.9716)	0.3200 (0.9208)	0.3251 (0.9537)	0.5222 (0.8593)	0.1502 (0.8995)	0.2967 (0.9385)	0.2685 (0.8850)	0.4815 (0.8610)
** (n,m) **	**CS**	**Boot-p**				**Boot-t**			
		** ${\hat{\alpha }}_{1}$ **	** ${\hat{\alpha }}_{2}$ **	** ${\hat{\beta }}_{1}$ **	** ${\hat{\beta }}_{2}$ **	** ${\hat{\alpha }}_{1}$ **	** ${\hat{\alpha }}_{2}$ **	** ${\hat{\beta }}_{1}$ **	** ${\hat{\beta }}_{2}$ **
(50,30)	L	0.1896 (0.9980)	0.2494 (1.0000)	0.1965 (1.0000)	0.2367 (1.0000)	0.1763 (1.0000)	0.3758 (1.0000)	0.2936 (0.9680)	0.6847 (1.0000)
	R	0.4031 (1.0000)	0.4247 (0.9700)	0.3794 (1.0000)	0.3844 (1.0000)	0.1949 (1.0000)	0.3362 (1.0000)	0.6204 (1.0000)	0.6925 (0.9980)
	U	1.1067 (1.0000)	0.8625 (0.9440)	0.9584 (1.0000)	0.8104 (0.9980)	0.2092 (1.0000)	0.3740 (1.0000)	0.4404 (1.0000)	0.6904 (0.9980)
	M	0.3577 (0.9920)	0.8454 (0.9620)	0.3591 (1.0000)	0.4814 (1.0000)	0.1723 (1.0000)	0.3511 (1.0000)	0.3122 (0.9920)	0.6078 (1.0000)
(80,30)	L	0.1667 (0.7280)	0.5349 (0.9960)	0.4662 (1.0000)	1.0506 (1.0000)	0.2643 (1.0000)	0.5976 (1.0000)	0.5443 (0.8400)	2.4375 (1.0000)
	R	0.3824 (0.9940)	0.4874 (1.0000)	0.8673 (0.9760)	0.8714 (0.9980)	0.3783 (1.0000)	0.4158 (1.0000)	1.7234 (1.0000)	0.9135 (1.0000)
	M	0.2411 (1.0000)	0.3769 (1.0000)	0.5051 (1.0000)	0.6723 (1.0000)	0.2782 (1.0000)	0.4712 (1.0000)	0.6343 (1.0000)	0.7061 (1.0000)
	U	0.1828 (0.9920)	0.5062 (1.0000)	0.5804 (1.0000)	1.2934 (1.0000)	0.2213 (1.0000)	0.4357 (1.0000)	0.3473 (1.0000)	0.5610 (1.0000)
(80,60)	L	0.1440 (1.0000)	0.1512 (0.9960)	0.1439 (0.9940)	0.1431 (1.0000)	0.1647 (0.9960)	0.2538 (1.0000)	0.2664 (1.0000)	0.4641 (0.9480)
	R	0.2703 (1.0000)	0.2325 (0.9940)	0.2635 (1.0000)	0.2511 (1.0000)	0.1426 (0.9920)	0.2374 (1.0000)	0.3219 (1.0000)	0.4766 (1.0000)
	M	0.2256 (0.9980)	0.3336 (1.0000)	0.2240 (0.9940)	0.2817 (1.0000)	0.1439 (0.9920)	0.2488 (1.0000)	0.2503 (0.9960)	0.4442 (0.9740)
	U	0.6082 (0.9260)	0.5932 (0.9980)	0.5061 (0.9960)	0.5153 (1.0000)	0.1318 (1.0000)	0.2446 (1.0000)	0.2715 (1.0000)	0.5292 (0.9920)

**Table A9:** ILs and CPs (within bracket) of Non-information Bayes HPD.

** (n,m) **	**CS**	** $T_{1}=0.8,T_{2}=1.2$ **				** $T_{1}=0.8,T_{2}=1.5$ **			
		** ${\hat{\alpha }}_{1}$ **	** ${\hat{\alpha }}_{2}$ **	** ${\hat{\beta }}_{1}$ **	** ${\hat{\beta }}_{2}$ **	** ${\hat{\alpha }}_{1}$ **	** ${\hat{\alpha }}_{2}$ **	** ${\hat{\beta }}_{1}$ **	** ${\hat{\beta }}_{2}$ **
(50,30)	L	0.2375 (0.8525)	0.4569 (0.8800)	0.8103 (0.9615)	0.9038 (0.9455)	0.2211 (0.8265)	0.4343 (0.8800)	0.7214 (0.9555)	0.8609 (0.9215)
	R	0.2096 (0.8985)	0.3860 (0.9180)	0.6890 (0.9545)	0.7627 (0.9205)	0.2056 (0.8950)	0.3782 (0.9165)	0.6613 (0.9555)	0.7511 (0.9230)
	U	0.2164 (0.8805)	0.4058 (0.9025)	0.7033 (0.9535)	0.7813 (0.9220)	0.2034 (0.8755)	0.3927 (0.9050)	0.6257 (0.9435)	0.7521 (0.9310)
	M	0.2399 (0.8890)	0.4413 (0.8930)	0.7347 (0.9595)	0.8060 (0.9240)	0.2214 (0.8840)	0.4222 (0.9015)	0.6583 (0.9480)	0.7769 (0.9315)
(80,30)	L	0.2416 (0.8405)	0.4678 (0.8675)	0.7989 (0.9365)	0.8845 (0.9050)	0.2232 (0.8425)	0.4477 (0.8730)	0.7036 (0.9440)	0.8366 (0.9025)
	R	0.2921 (0.9335)	0.4403 (0.8880)	0.7709 (0.9535)	0.7421 (0.9080)	0.2945 (0.9315)	0.4375 (0.8975)	0.7635 (0.9545)	0.7312 (0.9025)
	U	0.2224 (0.8935)	0.4070 (0.8910)	0.6369 (0.9465)	0.6803 (0.9010)	0.2155 (0.8935)	0.3972 (0.9150)	0.6162 (0.9400)	0.6671 (0.9235)
	M	0.2546 (0.8820)	0.4677 (0.8695)	0.6738 (0.9445)	0.7180 (0.8900)	0.2284 (0.8635)	0.4435 (0.8585)	0.6081 (0.9425)	0.6804 (0.9000)
(80,60)	L	0.1820 (0.8925)	0.3427 (0.9060)	0.5894 (0.9510)	0.6581 (0.9150)	0.1709 (0.8905)	0.3328 (0.9285)	0.5217 (0.9495)	0.6153 (0.9145)
	R	0.1602 (0.9235)	0.3020 (0.9255)	0.5180 (0.9405)	0.5884 (0.9160)	0.1515 (0.9080)	0.2902 (0.9275)	0.4559 (0.9405)	0.5495 (0.9105)
	U	0.1700 (0.9045)	0.3233 (0.9245)	0.5479 (0.9410)	0.6057 (0.9135)	0.1583 (0.8900)	0.3074 (0.9260)	0.4783 (0.9405)	0.5733 (0.9160)
	M	0.1786 (0.9120)	0.3303 (0.9330)	0.5585 (0.9410)	0.6122 (0.9130)	0.1656 (0.9150)	0.3175 (0.9240)	0.4958 (0.9460)	0.5836 (0.9175)
** (n,m) **	**CS**	** $T_{1}=1,T_{2}=1.5$ **				** $T_{1}=0.8,T_{2}=100$ **			
		** ${\hat{\alpha }}_{1}$ **	** ${\hat{\alpha }}_{2}$ **	** ${\hat{\beta }}_{1}$ **	** ${\hat{\beta }}_{2}$ **	** ${\hat{\alpha }}_{1}$ **	** ${\hat{\alpha }}_{2}$ **	** ${\hat{\beta }}_{1}$ **	** ${\hat{\beta }}_{2}$ **
(50,30)	L	0.2258 (0.8485)	0.4460 (0.8720)	0.7474 (0.9545)	0.8894 (0.9265)	0.2108 (0.8655)	0.4328 (0.9160)	0.3893 (0.9105)	0.7303 (0.8755)
	R	0.2070 (0.8905)	0.3836 (0.9215)	0.6836 (0.9505)	0.7691 (0.9295)	0.2085 (0.8790)	0.3886 (0.9235)	0.6841 (0.9405)	0.7702 (0.9295)
	U	0.2055 (0.8705)	0.4004 (0.9105)	0.6491 (0.9415)	0.7680 (0.9280)	0.1992 (0.8665)	0.3919 (0.9255)	0.5616 (0.9290)	0.7234 (0.9150)
	M	0.2222 (0.8705)	0.4331 (0.9025)	0.6727 (0.9465)	0.7955 (0.9080)	0.1987 (0.8670)	0.4081 (0.9360)	0.3992 (0.9040)	0.6692 (0.8900)
(80,30)	L	0.2252 (0.8285)	0.4469 (0.8895)	0.7056 (0.9370)	0.8493 (0.9090)	0.2067 (0.8540)	0.4339 (0.9215)	0.3746 (0.9055)	0.6992 (0.8625)
	R	0.2921 (0.9415)	0.4528 (0.9125)	0.7585 (0.9515)	0.7410 (0.9140)	0.2963 (0.9310)	0.4483 (0.9025)	0.7690 (0.9455)	0.7456 (0.9120)
	U	0.2161 (0.8970)	0.4010 (0.9170)	0.6103 (0.9455)	0.6594 (0.9100)	0.2147 (0.8970)	0.3997 (0.9120)	0.5954 (0.9430)	0.6632 (0.9270)
	M	0.2302 (0.8680)	0.4491 (0.8790)	0.6077 (0.9455)	0.6782 (0.9060)	0.1895 (0.8560)	0.4140 (0.9440)	0.3778 (0.9090)	0.5842 (0.8960)
(80,60)	L	0.1701 (0.9010)	0.3290 (0.9170)	0.5161 (0.9385)	0.6256 (0.9165)	0.1602 (0.8995)	0.3201 (0.9315)	0.2698 (0.8840)	0.5090 (0.8380)
	R	0.1507 (0.9115)	0.2914 (0.9345)	0.4585 (0.9480)	0.5528 (0.9185)	0.1463 (0.9050)	0.2849 (0.9435)	0.3958 (0.9370)	0.5183 (0.9145)
	U	0.1590 (0.9030)	0.3065 (0.9350)	0.4758 (0.9410)	0.5746 (0.9150)	0.1504 (0.9000)	0.2954 (0.9480)	0.3452 (0.9255)	0.5078 (0.9050)
	M	0.1644 (0.8950)	0.3170 (0.9235)	0.4944 (0.9490)	0.5837 (0.9135)	0.1509 (0.9105)	0.2993 (0.9320)	0.2784 (0.8865)	0.4891 (0.8680)

**Table A10:** ILs and CPs (within bracket) of Non-information Bayes HPD with ($T_{1}=100,T_{2}=101$).

(n,m)	CS	$T_{1}=100,T_{2}=101$			
		${\hat{\alpha }}_{1}$	${\hat{\alpha }}_{2}$	${\hat{\beta }}_{1}$	${\hat{\beta }}_{2}$
(50,30)	L	0.2130 (0.8510)	0.4367 (0.9185)	0.3871 (0.9195)	0.7260 (0.8855)
	R	0.2083 (0.9010)	0.3900 (0.9320)	0.6882 (0.9560)	0.7732 (0.9335)
	U	0.1995 (0.8685)	0.3959 (0.9255)	0.5065 (0.9270)	0.7224 (0.9090)
	M	0.1992 (0.8760)	0.4064 (0.9355)	0.3991 (0.9140)	0.6732 (0.8940)
(80,30)	L	0.2058 (0.8470)	0.4298 (0.9120)	0.3738 (0.9125)	0.7111 (0.8580)
	R	0.2959 (0.9295)	0.4513 (0.9115)	0.7622 (0.9525)	0.7429 (0.9125)
	U	0.2076 (0.8805)	0.3978 (0.9175)	0.5721 (0.9380)	0.6635 (0.9275)
	M	0.1880 (0.8390)	0.4167 (0.9415)	0.3829 (0.9115)	0.5822 (0.9250)
(80,60)	L	0.1599 (0.8975)	0.3176 (0.9295)	0.2694 (0.8845)	0.5138 (0.8250)
	R	0.1466 (0.8940)	0.2853 (0.9430)	0.3962 (0.9420)	0.5184 (0.9045)
	U	0.1509 (0.9060)	0.2964 (0.9420)	0.3260 (0.9205)	0.5029 (0.8955)
	M	0.1514 (0.9150)	0.2993 (0.9435)	0.2714 (0.8785)	0.4876 (0.8605)

**Table A11:** Acc and ESS for MCMC diagnostics under ($T_{1}=0.8,T_{2}=1.2$) and ($T_{1}=0.8,T_{2}=1.5$).

(n,m)	CS	$T_{1}=0.8,T_{2}=1.2$				$T_{1}=0.8,T_{2}=1.5$			
		Acc($\beta_{1}$)	Acc($\beta_{2}$)	ESS($\beta_{1}$)	ESS($\beta_{2}$)	Acc($\beta_{1}$)	Acc($\beta_{2}$)	ESS($\beta_{1}$)	ESS($\beta_{2}$)
Non-information Prior									
(50,30)	L	0.4425	0.4372	1708.26	1921.84	0.4094	0.4173	1823.28	1955.10
	M	0.3865	0.3991	1785.78	1799.15	0.3921	0.4320	1992.87	1950.68
	R	0.3881	0.3962	1936.73	1968.67	0.3678	0.4321	1895.66	2024.46
	U	0.4149	0.4464	1920.75	2018.97	0.3602	0.4450	2047.12	2022.59
(80,30)	L	0.4141	0.3889	1837.11	1877.12	0.3960	0.4645	1977.80	1820.50
	M	0.3254	0.3707	1414.75	1553.10	0.3680	0.3484	1737.41	1631.54
	R	0.3594	0.3551	1357.54	1479.47	0.3483	0.3440	1226.49	1531.61
	U	0.3208	0.3867	1624.16	1797.12	0.3511	0.3665	1846.57	1885.40
(80,60)	L	0.3438	0.3636	1907.81	2036.78	0.2968	0.3368	1834.86	1991.07
	M	0.3177	0.3522	1803.03	1951.02	0.2982	0.3455	1862.35	1968.81
	R	0.3165	0.3365	1865.19	1956.66	0.2780	0.3370	1709.99	1971.05
	U	0.3132	0.3325	1868.81	1911.91	0.2767	0.3305	1710.72	1932.38
Information Prior									
(50,30)	L	0.4370	0.4507	1801.53	1960.12	0.3658	0.4512	1929.21	1869.57
	M	0.3864	0.4442	1783.23	1913.05	0.3589	0.4095	1916.53	2034.98
	R	0.4086	0.3969	1887.45	1979.60	0.4016	0.3883	1978.25	2033.49
	U	0.4099	0.4446	1912.94	2014.72	0.3511	0.4033	1900.41	1957.96
(80,30)	L	0.4345	0.4347	1758.81	1959.44	0.3710	0.4285	1937.99	1908.05
	M	0.3295	0.3794	1473.45	1543.39	0.3594	0.3341	1699.92	1625.72
	R	0.3411	0.3588	1235.83	1486.27	0.3405	0.3578	1205.33	1548.12
	U	0.3286	0.3793	1677.77	1826.32	0.3504	0.3649	1794.85	1872.65
(80,60)	L	0.3210	0.3620	1874.83	1993.70	0.3193	0.3607	1889.33	2028.59
	M	0.3305	0.3388	1786.15	1875.92	0.3010	0.3352	1837.28	1935.11
	R	0.3079	0.3287	1888.94	1984.72	0.2914	0.3060	1755.86	1843.61
	U	0.3289	0.3476	1883.20	2000.60	0.2696	0.3523	1670.26	2063.04

**Table A12:** Acc and ESS for MCMC diagnostics under ($T_{1}=0.8,T_{2}=100.0$) and ($T_{1}=1.0,T_{2}=1.5$).

(n,m)	CS	$T_{1}=0.8,T_{2}=100.0$				$T_{1}=1.0,T_{2}=1.5$			
		Acc($\beta_{1}$)	Acc($\beta_{2}$)	ESS($\beta_{1}$)	ESS($\beta_{2}$)	Acc($\beta_{1}$)	Acc($\beta_{2}$)	ESS($\beta_{1}$)	ESS($\beta_{2}$)
Non-information Prior									
(50,30)	L	0.2043	0.3675	945.27	1607.83	0.3816	0.4664	1827.86	1974.78
	M	0.2437	0.3869	1454.76	2046.70	0.3441	0.4198	1827.10	1874.73
	R	0.3864	0.4078	1927.96	1915.62	0.3943	0.3959	1911.10	1988.03
	U	0.3130	0.3966	1690.60	2004.01	0.3642	0.4258	1961.84	2094.33
(80,30)	L	0.1915	0.3211	886.12	1673.11	0.3091	0.4649	1758.64	1842.71
	M	0.2396	0.3426	1539.24	1946.87	0.3355	0.3495	1612.89	1606.18
	R	0.3423	0.3746	1250.36	1495.00	0.3526	0.3940	1516.76	1690.80
	U	0.3235	0.3345	1798.22	1867.83	0.3523	0.3337	1931.45	1811.05
(80,60)	L	0.1457	0.2674	663.92	1345.65	0.3349	0.3644	1899.77	2051.58
	M	0.1603	0.2793	929.59	1651.29	0.2777	0.3479	1732.47	2036.77
	R	0.2368	0.2902	1373.83	1764.34	0.2725	0.3245	1701.41	1908.37
	U	0.2069	0.2871	1143.62	1713.10	0.2790	0.3300	1743.71	1927.67
Information Prior									
(50,30)	L	0.2078	0.3525	1002.50	1725.10	0.4019	0.4061	1969.58	1942.02
	M	0.2401	0.3620	1328.53	1936.13	0.3252	0.4350	1816.45	1927.17
	R	0.3488	0.3819	1750.06	2034.00	0.3659	0.3888	1898.98	1899.89
	U	0.3135	0.3916	1791.34	1997.60	0.3606	0.3979	1963.50	2008.02
(80,30)	L	0.2151	0.3377	1037.03	1819.38	0.3751	0.4153	1950.19	1949.04
	M	0.2190	0.3376	1365.41	1932.77	0.3282	0.3491	1696.93	1697.44
	R	0.3382	0.3525	1362.21	1559.86	0.3523	0.3858	1417.75	1623.20
	U	0.3474	0.3766	1784.51	1923.07	0.3045	0.3829	1663.47	1902.09
(80,60)	L	0.1450	0.2697	721.34	1438.20	0.3113	0.3513	1872.97	1943.56
	M	0.1579	0.2743	841.22	1614.34	0.2890	0.3661	1771.50	2095.96
	R	0.2246	0.3013	1300.02	1769.85	0.2886	0.3126	1720.47	1918.42
	U	0.1906	0.2796	995.06	1558.30	0.2834	0.3199	1751.19	1944.97

**Table A13:** Acc and ESS for MCMC diagnostics under ($T_{1}=100,T_{2}=101$) with Non-information and Information Priors side by side.

(n,m)	CS	Non-Information Prior				Information Prior			
		Acc($\beta_{1}$)	Acc($\beta_{2}$)	ESS($\beta_{1}$)	ESS($\beta_{2}$)	Acc($\beta_{1}$)	Acc($\beta_{2}$)	ESS($\beta_{1}$)	ESS($\beta_{2}$)
(50,30)	L	0.2133	0.3513	1027.86	1775.79	0.1888	0.3735	942.00	1587.13
	M	0.2303	0.3814	1345.84	1987.05	0.2268	0.3519	1316.68	1984.58
	R	0.3558	0.4142	1917.75	2051.24	0.3657	0.4089	1905.58	2046.95
	U	0.2861	0.4047	1597.61	2093.16	0.3070	0.3863	1687.93	2015.13
(80,30)	L	0.2118	0.3499	1096.60	1722.37	0.1969	0.3447	1057.60	1690.76
	M	0.2314	0.3503	1490.42	2077.79	0.2425	0.3381	1565.49	2034.29
	R	0.3188	0.3753	1282.80	1562.33	0.3616	0.3608	1329.25	1590.58
	U	0.3558	0.3536	1730.40	1812.58	0.3272	0.3410	1825.40	1860.48
(80,60)	L	0.1492	0.2924	724.42	1397.43	0.1473	0.2799	728.37	1379.58
	M	0.1561	0.2862	858.84	1684.65	0.1540	0.2718	878.55	1656.39
	R	0.2190	0.2982	1293.99	1765.39	0.2294	0.3177	1302.14	1779.96
	U	0.1853	0.3109	1002.80	1623.39	0.1951	0.2878	1113.39	1696.28
